# Robotic resection of a rudimentary horn pregnancy with the Da Vinci system: first case and systematic review of published cases in the 21st century

**DOI:** 10.3389/fsurg.2026.1757761

**Published:** 2026-05-18

**Authors:** A. Favre-Inhofer, P. Molnar, L. Jung, M. Hübner, M. Medl, C. Jäger, I. Juhasz-Böss, S. Huwer

**Affiliations:** Klinik für Frauenheilkunde, Medizinische Fakultät, Albert-Ludwigs-Universität Freiburg, Freiburg, Germany

**Keywords:** ectopic pregnancy, minimal invasive surgery (MIS), robotic surgery, rudimentary horn pregnancy, systematic review, uterine anomaly

## Abstract

**Background:**

Rudimentary horn pregnancy is a rare, high-risk ectopic gestation associated with significant maternal and fetal morbidity. Advances in minimally invasive surgery have expanded management options, but robotic-assisted resections have not been previously reported.

**Objectives:**

This study aims to present the first robotic resection of a non-communicating rudimentary horn pregnancy and systematically review all published cases from 2000 onward to summarize diagnostic and management outcomes.

**Data sources:**

A systematic literature search was conducted in PubMed up to August 14, 2025.

**Eligibility criteria:**

We included English-language case reports and series of non-communicating rudimentary horn pregnancies published from 2000 onward.

**Methods:**

Data on patient characteristics, diagnostic modalities, management strategies, and maternal-fetal outcomes were extracted following PRISMA 2020 guidelines.

**Results:**

The review identified 187 cases across 163 publications. Most pregnancies were diagnosed intraoperatively, with rupture occurring in 44%—predominantly during the second trimester—and a maternal mortality rate of 1.2%. Placenta accreta spectrum was reported in 39% of cases. Minimally invasive procedures, including our published robotic approach, are increasingly used in selected stable patients.

**Limitations:**

The evidence is based primarily on retrospective case reports, which are prone to publication bias and heterogeneity.

**Conclusions:**

Rudimentary horn pregnancy poses significant diagnostic and surgical challenges with high risk of rupture. Early recognition and minimally invasive management—including robotic surgery—may improve outcomes. Continued systematic reporting is critical to guide clinical practice.

## Introduction

The unicornuate uterus is a rare Müllerian developmental anomaly, estimated to occur in approximately 1 in 500 women and accounting for 4%–10% of all uterine malformations (1). A pregnancy within a rudimentary horn is even more exceptional, with an incidence ranging between 1 in 76,000 and 1 in 150,000. These pregnancies typically result from transperitoneal migration of sperm or a fertilized ovum to the contralateral side, allowing implantation within the non-communicating horn.

The diagnosis of rudimentary horn pregnancy poses a considerable challenge owing to its rarity and nonspecific clinical presentation. In many cases, the condition is only identified at the time of surgery. Advances in imaging (MRI) have improved diagnostic possibilities (2). Despite these advances, a delayed or missed diagnosis continues to carry significant risks for maternal and fetal health.

Historically, maternal outcomes were especially poor. Maternal mortality reached nearly 10% in the first half of the 20th century, but improvements in surgical techniques and earlier recognition have since reduced this figure to around 1.2%. Mortality remains closely linked to horn rupture and subsequent hemorrhagic shock. Fetal outcomes remain unfavorable: survival rates throughout the 20th century were reported at only 6% (3). Since the systematic review by Tellum et al., no systematic review has analyzed all pregnancies in non-communicating rudimentary horns, including their clinical presentation, diagnosis, treatment, and outcomes.

In this article, we present the first reported case of robotic-assisted resection of a rudimentary horn pregnancy. Furthermore, we provide a systematic review of all non-communicating rudimentary horn pregnancies cases published since 2000, conducted according to the PRISMA 2020 guidelines (4), and supplement this with a comprehensive overview of previously reported cases from earlier decades.

## Material and methods

### Case description

We present a case of non-communicating rudimentary horn pregnancy managed with Da Vinci Xi robotic surgery, with written informed consent obtained for publication.

### Protocol and registration

This systematic review followed PRISMA 2020 guidelines. The review protocol and full search details are available in the Supplementary Appendix.

### Eligibility criteria

Eligible studies were original reports of non-communicating rudimentary horn pregnancies. Exclusion criteria comprised non-English publications, unavailable full texts, non-pertinent uterine anomalies, cases not involving pregnancy, or irrelevant clinical conditions. Two eras (pre-2000 and post-2000) were analyzed separately.

### Information sources and search strategy

**Figure 1:**
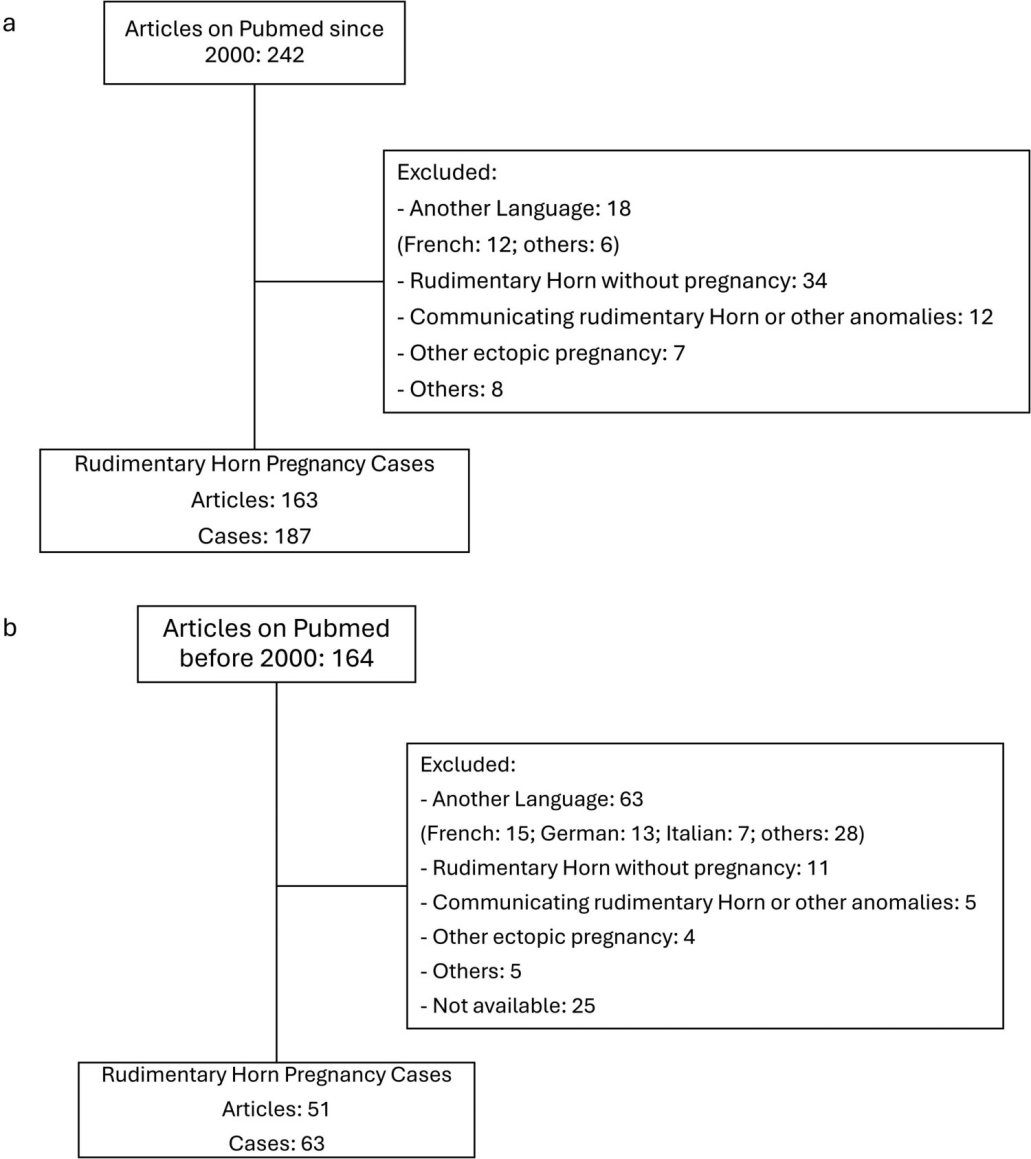
Flowchart of article identification, screening, and inclusion for published cases of rudimentary horn pregnancy before **(b)** and after 2000 **(a).**

PubMed was systematically searched [“rudimentary horn pregnancy” (All Fields) NOT “cornual” (All Fields)] on July 12 and August 14, 2025, without language or publication filters. Search dates, strategy, and reasons for exclusion are detailed in the appendix and PRISMA flowchart (Figure 1).

### Selection process

Titles and abstracts were screened for eligibility; eligible articles underwent full-text assessment. Reasons for exclusion at each stage were documented.

### Data collection process and data items

Data were extracted independently into standardized tables, then harmonized. Extracted items included publication year, country, demographics, gestational age, fetal cardiac activity, anatomical anomalies, clinical presentation, diagnostic method, treatment, uterine rupture or placenta accreta, horn characteristics, estimated blood loss, fetal outcome, and case remarks. Missing data were excluded from synthesis.

### Risk of bias and certainty assessment

Risk of bias was evaluated for each included report using adapted case report criteria (clinical completeness, diagnostic confirmation, and outcome reporting). Certainty of evidence was rated low due to reliance on case reports and potential reporting bias.

### Effect measures and synthesis methods

Dichotomous outcomes were presented as proportions or risk ratios; continuous variables were presented as mean differences. Fisher's exact test and Student's *t*-test were used for group comparisons; the significance threshold was *p* < 0.05.

## Results

### Case presentation

A 30-year-old woman was transferred to our department with a suspected extrauterine pregnancy at 5 weeks of gestation. Her medical history included multiple reconstructive surgeries for anal atresia at birth, right ovarian cystectomy via laparotomy during adolescence, and congenital left renal agenesis. On admission, she was hemodynamically stable and asymptomatic, reporting no abdominal pain or vaginal bleeding. Serum β-hCG was 18,000 IU/L.

Review of prior surgical records confirmed a unicornuate uterus (U4a, ESHRE/ESGE classification) (5). The right uterine horn communicated with the cervix and vagina and was associated with normal adnexa. The left rudimentary horn was completely separated, lacked any vaginal communication, and had normal left adnexa.

Transvaginal ultrasound revealed a viable intrauterine-appearing gestational sac corresponding to 5 weeks' gestation located within the left rudimentary horn, distinct from the right uterine cavity. No free intraperitoneal fluid was noted.

Following multidisciplinary counseling regarding the diagnosis and associated risk of rupture, a stepwise management plan was implemented. The patient received intramuscular methotrexate for trophoblastic suppression, followed by robotic-assisted laparoscopic excision of the left rudimentary horn. A concurrent diagnostic hysteroscopy and chromopertubation of the right uterine horn were also performed to assess uterine configuration and tubal patency for future fertility planning.

Hysteroscopy demonstrated a tubular right unicornuate horn with a single right tubal ostium. Robotic surgery was performed using the Da Vinci Xi platform. Abdominal access was established via Veress needle insufflation at the infraumbilical site. Due to dense right-sided adhesions from prior surgery, all trocars were positioned along the left abdominal side, including three robotic arms (30° camera, monopolar curved scissors, and fenestrated bipolar forceps) and an assistant trocar at Palmer's point.

Intraoperatively (Figure 3), the right communicating horn was identified beneath the peritoneum. The right ovary was obscured by adhesions; however, the right tube was patent on chromopertubation. The left rudimentary horn, entirely separate from the right, contained the gestational sac and normal left adnexa. Excision of the rudimentary horn and left fallopian tube was completed through careful dissection and division of the left utero-ovarian ligament, round ligament, and uterine artery, without any anatomical connection to the contralateral horn. The specimen was retrieved via Palmer's point.

Estimated blood loss was minimal, and postoperative recovery was uneventful. Serum β-hCG levels declined rapidly. Histopathological examination of the excised specimen confirmed the presence of chorionic villi and decidualized endometrium, consistent with an early intrauterine gestation within a non-communicating rudimentary horn.

### Systematic review

#### Results of study selection

**Figure 2:**
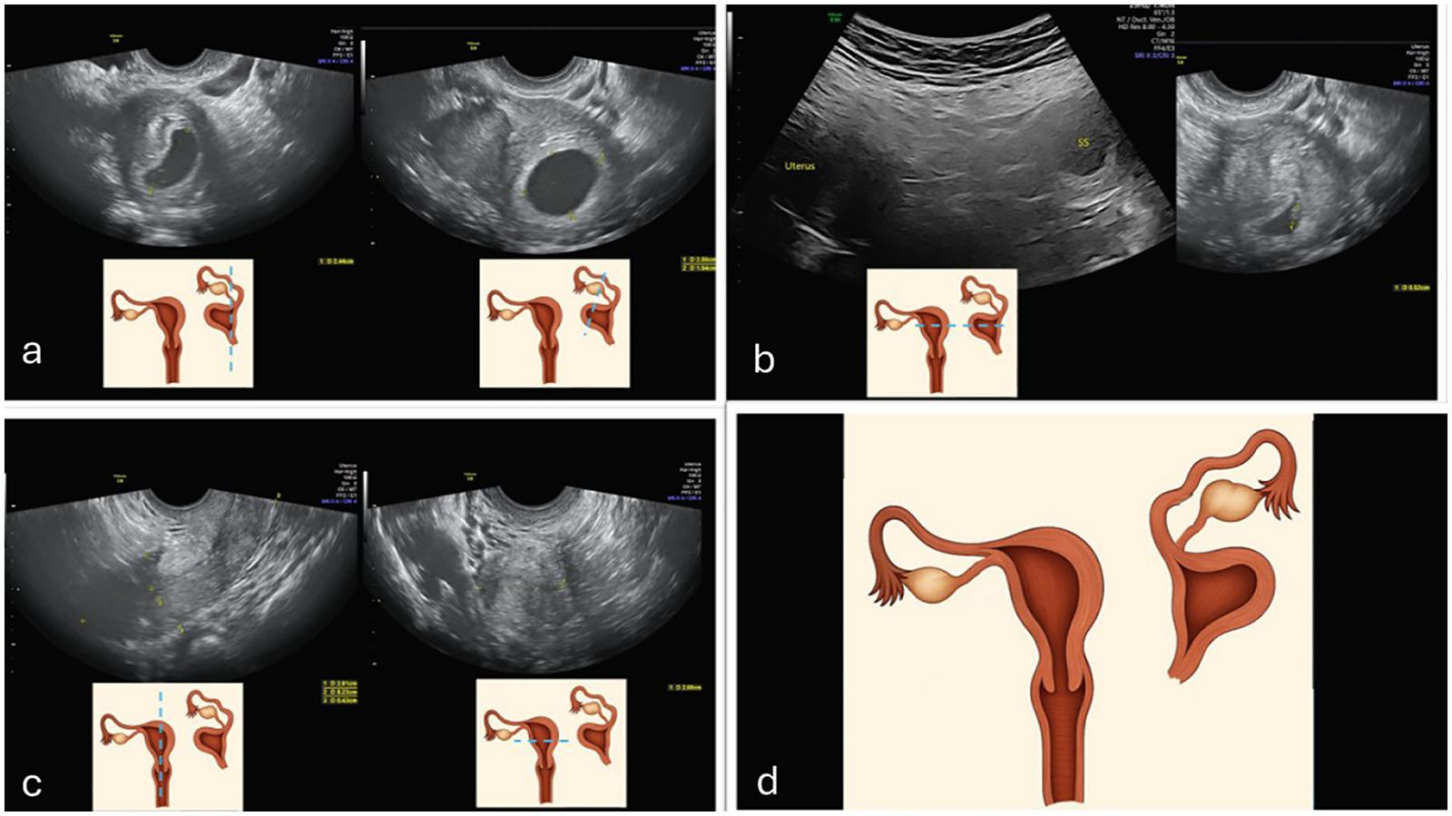
Transvaginal and abdominal ultrasound imaging of right rudimentary horn pregnancy **(a,b)** and left unicornuate horn **(c)**; schematic representation of the anatomic anomaly **(d)**.

For the period prior to 2000 (Figure 2b), the search identified 164 publications. Of these, 113 were excluded.

**Figure 3:**
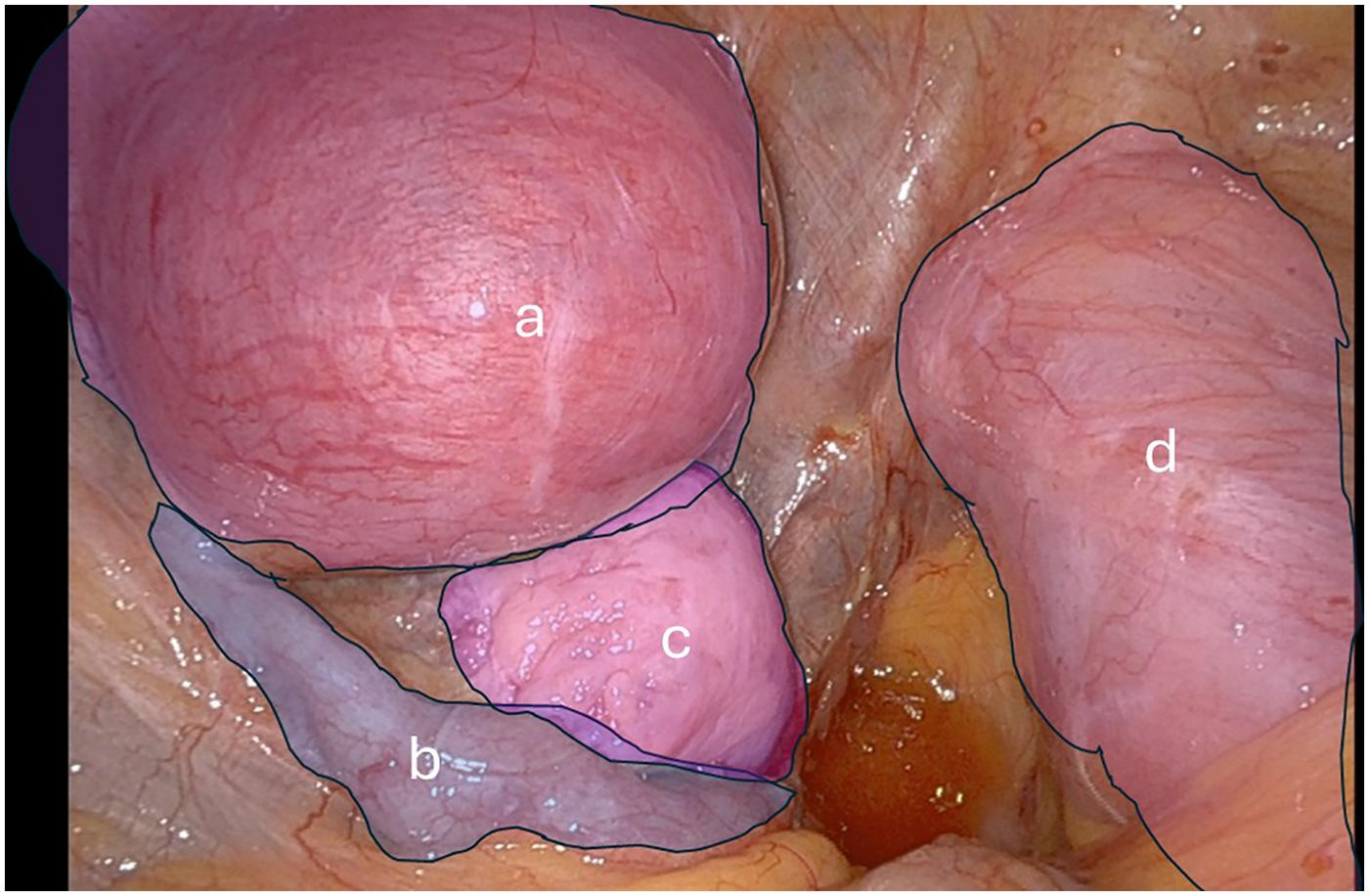
Pelvic intraoperative view: **(a)** left rudimentary horn pregnancy; **(b)** left fallopian tube; **(c)** left ovary; **(d)** right unicornuate horn.

For the period from 2000 onward (Figure 2a), the search yielded 242 publications. After screening, 79 articles were excluded.

The complete table is available in the appendix and includes all DOI references; if no DOI was available, a PubMed Identifier (PMID) was used to identify the article from which the data were extracted.

### Patient and anatomic characteristics

The mean age of patients was 26.9 years in the overall cohort (Table 1). Before 2000, the study population was slightly younger, with a mean age of 25 years compared with 27 years after 2000 (*p* = 0.024). For 45% of patients, this was their first pregnancy, while 41% had already delivered a child. Associated anomalies, most often renal agenesis on the same side as the rudimentary horn, were present in 25% of cases. However, only 71 cases (28%) documented the presence or absence of anomalies. The rudimentary horn was most frequently right-sided (62%). The anatomical type of connection between the rudimentary horn and the unicornuate uterus was a broad muscular attachment (75%), a thin fibrous band (21%), or no connection at all (3%). Abnormal placentation was described in 33 of 72 (46%) cases with available data.

**Table 1:** Comparison of patient demographics and anatomic characteristics of non-communicating rudimentary horn pregnancies before and after 2000.

Patients and anatomical characteristics		After 2000 (*N* = 187)	Before 2000 (*N* = 63)	p_value	All of the cohorte (*N* = 250)
Age		**27 years**	**25 years**	**0**.**024**	26,9 years
First gravidity Uni- or Multipara		78/179 (44%) **100/172 (58%)**	29/58 (50%) **23/58 (40%)**	0.45 **0.016**	107/237 (45%) 95/135 (41%)
Associated anatomic anomalies		**11/56 (20%)**	**7/15 (47%)**	**0**.**046**	18/71 (25%)
Right-sided rudimentary horn		111/175 (63%)	33/50 (66%)	0.87	134/216 (62%)
Type of anatomical connection between rudimentary and unicornuate horn	Broad muscular attachment Thin fibrous band No connection	88/118 (75%) 25/118 (21%) 5/118 (4%)	29/37 (78%) 8/37 (22%) 0	0.83 1.0 1.0	117/155 (75%) 33/155 (21%) 5/155 (3%)
Placenta accreta		21/54 (39%)	12/18 (67%)	0.057	33/72 (46%)

The bold values indicate statistically significant results.

### Rudimentary horn ruptures

**Figure 4:**
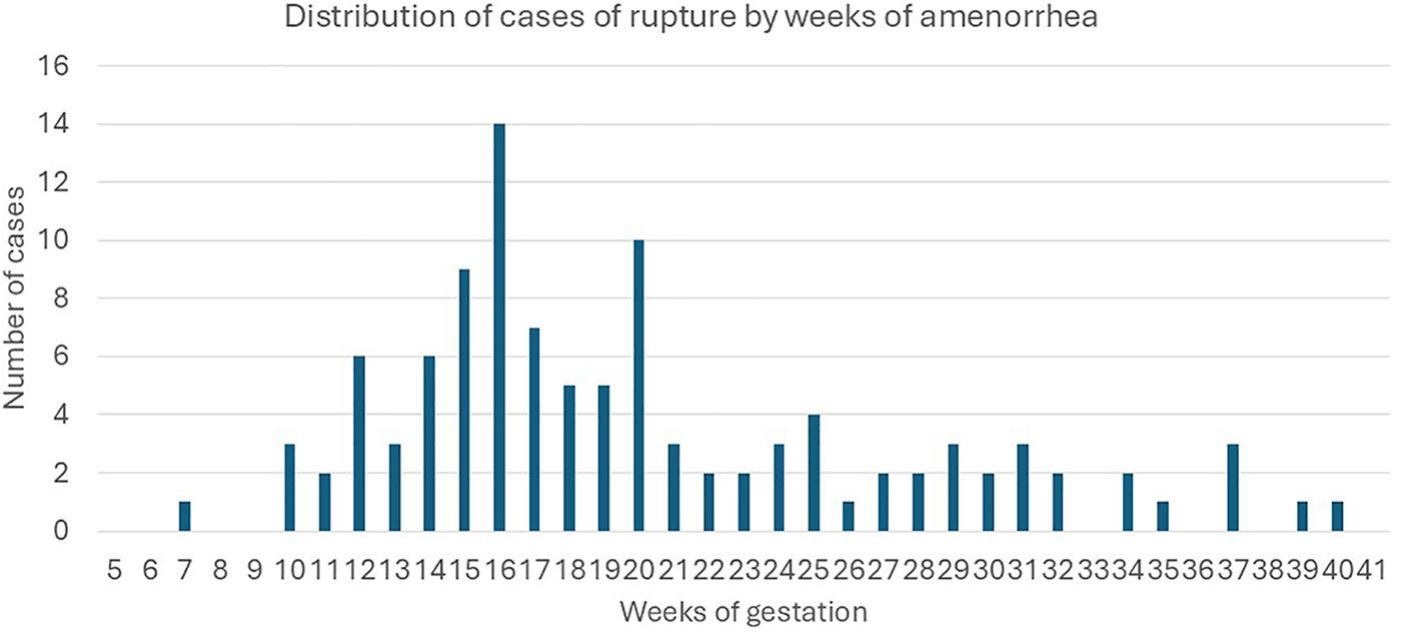
Distribution of the cases of rupture by weeks of amenorrhea.

Figure 4 shows the distribution of rupture cases by gestational age (weeks of amenorrhea). Most ruptures occurred in the second trimester, with a peak between weeks 16 and 20.

When comparing rupture vs. non-rupture cases after 2000 (Table 2), ruptures occurred predominantly in the second trimester (69% vs. 24% without rupture; *p* < 0.01). There was no significant difference regarding the presence of intrauterine cardiac activity (57% with rupture vs. 65% without rupture; *p* = 0.4). Patients experiencing a rudimentary horn rupture were more symptomatic: 94% had abdominal pain (vs. 25% without rupture; *p* < 0.01), 29% presented with vomiting (vs. 2%; *p* < 0.01), and 70% were in hypovolemic shock (vs. 1%; *p* < 0.01). Vaginal bleeding did not differ between groups. The mean blood loss in rupture cases was 2,200 mL (vs. 200 mL without rupture), and massive bleeding (>1 L) occurred in 91% of rupture cases (vs. 7%; *p* < 0.01). Blood transfusion was required in 82% of rupture cases compared with 6% in non-rupture cases (*p* < 0.01). Surgical treatment was performed via laparotomy in 92% of rupture cases vs. 49% without rupture (*p* < 0.01). Conversely, laparoscopy was used in 6% of rupture cases compared with 48% without rupture (*p* < 0.01).

**Table 2:** Clinical presentation, management, and outcomes of non-communicating rudimentary horn pregnancies with and without uterine rupture before and after 2000.

Clinical presentation, management, and outcome	After 2000 (*N* = 187)			Before 2000 (*N* = 63)			All cohort (*N* = 250)		
	Rupture (*N* = 83)	No Rupture (*N* = 104)	*p*	Rupture (*N* = 26)	No Rupture (*N* = 37)	*p*	Rupture (*N* = 109)	No Rupture (*N* = 141)	*p*
Trimester of Pregnancy First trimester Second trimester Third trimester	**12/83 (14%)** **57/83 (69%)** 14/83 (17%)	**60/104 (58%)** **25/104 (24%)** 19/104 (18%)	**<0.01** **<0.01** 0.85	3/25 (12%) 17/26 (65%) 6/25 (23%)	10/37 (27%) 15/37 (40%) 12/37 (32%)	0.21 0.07 0.57	**15/109 (14%)** **74/109 (68%)** 20/109 (18%)	**70/141 (50%)** **40/141 (28%)** 31/141 (22%)	**<0.01** **<0.01** 0.53
Heart activity Present	42/73 (57%)	49/75 (65%)	0.40	9/14 (64%)	14/27 (52%)	0.52	51/87 (59%)	63/102 (62%)	0.77
Symptoms Pain Vomiting Hpovolemic shock Vaginal Bleeding	**76/80 (94%)** **18/62 (29%)** **57/80 (70%)** 6/77 (8%)	**26/103 (25%)** **2/101 (2%)** **1/102 (1%)** 11/102 (11%)	**<0.01** **<0.01** **<0.01** 0.61	**24/26 (92%)** **8/19 (42%)** **20/26 (77%)** 3/22 (14%)	**11/35 (31%)** **1/33 (3%)** **0/34 (0%)** 4/34 (12%)	**<0.01** **<0.01** **<0.01** **1**	**100/106 (94%)** **26/81 (32%)** **77/106 (73%)** 9/99 (9%)	**37/138 (27%)** **3/134 (2%)** **1/136 (0,7%)** 15/136 (11%)	**<0.01** **<0.01** **<0.01** 0.67
Massive bleeding Mean Blood Loss (mL) Transfusion	**62/68 (91%)** **2,200** **55/67 (82%)**	**4/57 (7%)** **200** **5/87 (6%)**	**<0.01** **<0.01** **<0.01**	**18/21 (86%)** **1,943** **14/15 (93%)**	**0/7 (0%)** **Minimal** **0/14 (0%)**	**<0.01** **<0.01** **<0.01**	**80/89 (90%)** **2,146** **69/82 (84%)**	**4/64 (6%)** **201** **5/101 (4%)**	**<0.01** **<0.01** **<0.01**
Treatment Laparotomy Conversion to laparotomy Laparoscopy	**76/83 (92%)** 6/83 (7%) **5/83 (6%)**	**50/102 (49%)** 6/102 (7%) **49/102 (48%)**	**<0.01** 0.77 **<0.01**	23/23 (100%) 0 0	32/36 (92%) 1/36 (3%) 4/36 (11%)	0.15 1 1	**99/106 (93%)** 6/83 (7%) **5/83 (6%)**	**82/138 (59%)** 7/138 (5%) **53/138 (38%)**	**<0.01** 0.56 **<0.01**
Living Infant	15/83 (18%)	14/104 (13%)	0.42	6/26 (23%)	7/37 (19%)	0.75	21/109 (28%)	21/141 (15%)	0,40

The bold values indicate statistically significant results.

### Treatment and diagnosis

**Figure 5:**
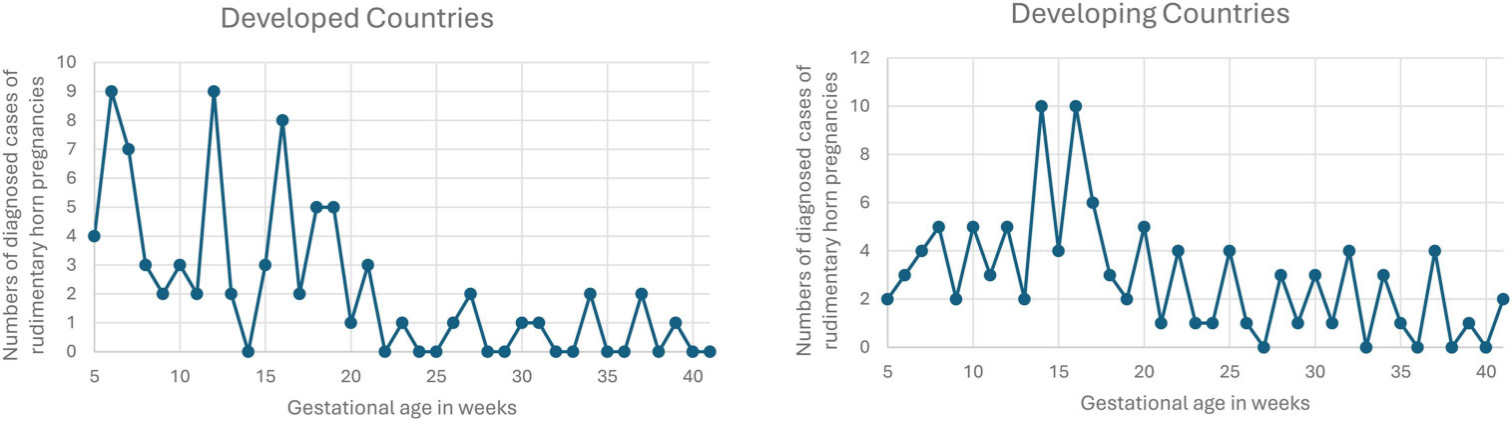
Diagnosis of rudimentary horn pregnancy by gestational age in developed and developing countries.

When comparing patient characteristics, diagnosis, and treatment between developed and developing countries as defined by the OECD (6), no difference was found regarding primigravida vs. multigravida patients (Table 3). Patients in developing countries were younger (26.2 years) than those in developed countries (28.6 years; *p* = 0.01). Diagnosis occurred earlier in developed countries, with 51% identified in the first trimester vs. 29% in developing countries (*p* < 0.01). In contrast, third-trimester cases were more frequent in developing countries (24%) compared with developed countries (9%; *p* < 0.01). Consequently, rupture rates were higher in developing countries (54%) vs. developed countries (32%; *p* < 0.01). Figure 5 illustrates the timing of diagnosis by gestational week in both settings. Early diagnosis (before 20 weeks) was more frequent in developed countries, while in developing countries diagnosis often occurred around 15 weeks and was reported equally in the first and third trimesters.

**Table 3:** Comparison of patient characteristics, diagnosis, treatment, and outcomes of non-communicating rudimentary horn pregnancies in developed and developing countries (after 2000).

Clinical presentation, management, and outcome	Age	26,6 years	28,6 years	26,2 years	**0,01**
Patients characteristics	First gravidity	78/184 (42%)	35/77 (45%)	43/107 (40%)	0.55
	Uni- or Multipara	84/184 (46%)	32/77 (42%)	52/107 (49%)	0.37
	Rupture	**83/187 (44%)**	**25/79 (32%)**	**58/108 (54%)**	**<0.01**
	First trimester	**71/187 (38%)**	**40/79 (51%)**	**31/108 (29%)**	**<0.01**
	Second trimester	83/187 (44%)	32/79 (41%)	51/108 (47%)	0.38
	Third trimester	**33/187 (18%)**	**7/79 (9%)**	**26/108 (24%)**	**<0.01**
Diagnosis	Diagnosed by Ultrasound	54/187 (29%)	26/79 (33%)	28/108 (26%)	0.33
	MRI performed	**58/187 (31%)**	**34/79 (43%)**	**24/108 (22%)**	**<0.01**
	Diagnosis by MRI	44/58 (76%)	27/34 (79%)	17/24 (71%)	0.54
	Operative Diagnosis	**113/187 (60%)**	**40/79 (51%)**	**73/108 (68%)**	**0**.**023**
Treatment	Methotrexate	11/154 (7%)	8/72 (11%)	3/82 (4%)	0.11
	Methotrexate and laparoscopy	8/154 (5%)	6/72 (8%)	2/82 (2%)	0.15
	Laparotomy only	**81/154 (53%)**	**28/72 (39%)**	**53/82 (65%)**	**<0.01**
	Laparoscopy only	**47/154 (31%)**	**27/72 (38%)**	**20/82 (24%)**	**0**.**083**
	Conversion to Laparotomy	12/154 (8%)	8/72 (11%)	4/82 (5%)	0.23
Fetal outcome	Living infant	28/186 (15%)	8/79 (10%)	20/107 (19%)	0.15

The bold values indicate statistically significant results.

Diagnostic performance of ultrasound and MRI was similar in both regions (Table 3). Ultrasound diagnosed 29% of rudimentary horn pregnancies overall. When performed, MRI identified 76% of cases but was less frequently used in developing countries (22%) compared with developed countries (43%; *p* < 0.01). Diagnosis during surgery was more frequent in developing countries (68%) compared with developed countries (51%, *p* = 0.02), highlighting that diagnosis was made before surgery in only half of all cases.

Methotrexate therapy was rare (7% of cases) and showed no difference between developed and developing countries (*p* = 0.11). Laparoscopic management was more common in developed countries (38% vs. 24%, *p* = 0.08), while laparotomy was more frequent in developing countries (65% vs. 39%, *p* < 0.01). No difference could be established in live birth outcomes due to the very small number of cases (15%; *p* = 0.15).

**Figure 6:**
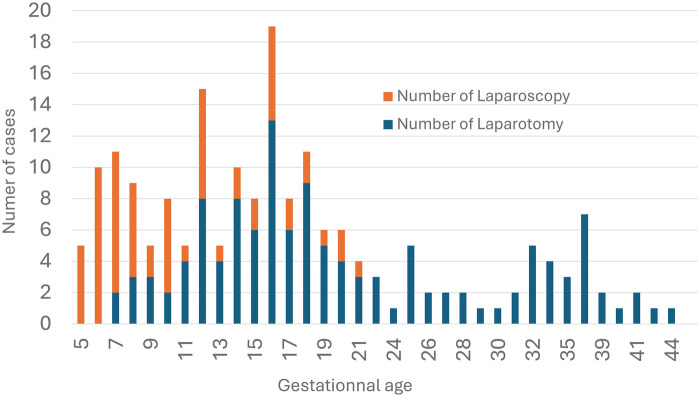
Laparoscopic and laparotomic management of rudimentary horn pregnancy by gestational age.

Figure 6 shows laparoscopic vs. laparotomic management according to gestational age. In weeks 5 and 6, all treatments were laparoscopic. Between weeks 7 and 10, laparoscopy was predominant, with only a few published cases in the second trimester. The latest laparoscopic procedure reported was at 19 weeks (7).

### Rare cases within this rare pathology

Since 2000, three maternal deaths (8–10) were reported, giving a maternal mortality rate of 1.2%. These were autopsy diagnoses: one in 2013 in Japan (8), where a 30-year-old gravida presented with abdominal pain at 19 weeks and died shortly after admission from hemorrhagic shock; one in 2018 in Tunisia (10), where a 35-year-old primigravida died at 16 weeks after presenting with abdominal pain; and one in 2023 in South Africa (9), where a 29-year-old gravida 2, para 1 died at home following abdominal pain.

Two cases of lithopedion were described, a rare condition in which a dead fetus calcifies within the maternal body. The first, reported in 2003 (11), described a 5-year lithopedion at 37 weeks, diagnosed after perforation of the sigmoid colon by fetal bone, presenting as abdominal pain and fever. The second case, reported in 2017 (12), involved a 36-year-old woman with chronic pelvic pain and infertility. CT revealed a 12 cm right latero-uterine mass, with femoral length consistent with a full-term fetus from a pregnancy 18 years prior.

Three cases of rudimentary horn pregnancy with concomitant torsion were documented (13–15). The first, in India in 2015 (13), was diagnosed at 20 weeks and treated laparoscopically. The second, in India in 2018 (14), involved a 17-week pregnancy diagnosed after fetal demise and failed induction, leading to rupture and torsion. The third, in Ethiopia in 2024 (15), reported a combined rupture and torsion at 32 weeks.

Six twin pregnancies were documented. Two heterotopic cases (16, 17) presented with rudimentary horn rupture at 12 and 15 weeks, with the twins in the unicornuate uterus delivered at term. Another heterotopic pregnancy (18) was managed by elective Cesarean delivery at 35 weeks, with both twins surviving. One case of rudimentary horn rupture at 15 weeks (19) involved monochorionic twins, one acardiac. Another heterotopic pregnancy (20) presented with horn rupture at 17 weeks, followed by termination of the unicornuate pregnancy due to fetal anomaly. One case (21) involved severe preeclampsia at 35 weeks, with a Cesarean delivery during which a rudimentary horn pregnancy was discovered.

A single triplet pregnancy was reported (22), following clomiphene citrate induction. This right-sided rudimentary horn pregnancy ruptured at 20 weeks.

One case of invasive hydatidiform mole developing in a right rudimentary horn was reported (23), managed laparoscopically.

Finally, three cases of rudimentary horn rupture leading to secondary abdominal pregnancy were reported from India (24–26). In each case, the covered rupture allowed placental attachment to the rudimentary horn with ongoing fetal blood supply. All three pregnancies resulted in live births at term but required extensive surgical management.

## Discussion

### Rarity and epidemiologic context

To our knowledge, this is the first published case of a laparoscopic robotic resection of a rudimentary horn pregnancy. Two robotic resections of rudimentary horns have previously been reported (27, 28), but in those cases the patients were not pregnant. Since 1998, when Yahata et al. (29) published the first laparoscopic resection, many laparoscopic resections have been performed worldwide.

Since Nahum GG (3) identified 588 cases of rudimentary horn pregnancy in the 20th century, only one publication (30) has systematically reviewed rudimentary horn pregnancies since 2000. Our systematic review identified 163 articles since 2000, compiling 187 cases of non-communicating rudimentary horn pregnancies.

### Incidence and laterality of uterine anomalies

The unicornuate uterus is a rare anomaly occurring in 0.5% of women (31), of whom 84% have a rudimentary horn. Since 2000, 63% of rudimentary horns were right-sided, which is significantly higher than expected (*p* < 0.01). Associated anatomical anomalies were reported in 20% of cases, usually on the same side as the rudimentary horn. However, only one-third of reports specified whether additional anomalies were present. The incidence of renal anomalies in women with a unicornuate uterus varies in the literature from 40% (32) to 60% (33). In all cases, including ours, the anomalies were ipsilateral to the rudimentary horn. The predominance of right-sided anomalies remains unclear. The developmental asymmetry theory suggests that early differences in embryonic gene expression cause unequal teratogen sensitivity, while the vascular supply theory proposes that the right side is more prone to vascular injury, especially in animal models, leading to more frequent anomalies (34). These findings support theories of embryologic asymmetry and vascular vulnerability as potential explanations for the right-sided predominance and ipsilateral renal defects observed in these patients.

### Placenta accreta spectrum

Since 2000, placenta accreta has been reported in 39% of rudimentary horn pregnancies. In the general population, the risk of placenta accreta ranges from 1 in 533 to 1 in 730 deliveries (35). Placenta accreta spectrum in an unscarred uterus is rare but may be explained in rudimentary horns by myometrial and decidual deficiency and abnormal vascularization. Oral et al. (36) reported an incidence of 11.9% (21.2% when ruptured). Although our incidence may be overestimated due to incomplete reporting, it is clear that structural and vascular deficiencies inherent to the rudimentary horn markedly increase susceptibility to the placenta accreta spectrum.

### Rupture and maternal outcomes

Rupture of a rudimentary horn pregnancy most frequently occurred between 16 and 20 weeks of gestation, with 69% of ruptures in the second trimester. In our series after 2000, the rupture rate was 44%, consistent with the literature (37); in the 20th century, the rate was 47% (3). Rupture is a life-threatening complication. Ectopic pregnancy remains an important cause of maternal mortality, particularly in the first trimester and in low-resource settings, where mortality rates range from 1%–3% compared to 0.1%–0.3% in developed countries (38). The overall mortality rate for rudimentary horn pregnancy is 1.2%, consistent with Nahum GG (3). Among patients with rupture, 94% presented with abdominal pain, 70% with hypovolemic shock, massive hemorrhage occurred in 91%, and transfusion was required in 86%. Surgical management since 2000 has been laparotomy in 92% of cases. A laparoscopic approach was feasible only in five hemodynamically stable patients (30, 39–41). These data emphasize that timely diagnosis before rupture remains crucial, as the condition continues to carry significant maternal risk despite surgical advances.

### Diagnosis and imaging modalities

Diagnosis of rudimentary horn pregnancy remains intraoperative in about 60% of cases. Only 29% are diagnosed by ultrasound, consistent with previous reports (30). Ultrasound sensitivity was calculated at 26% in one analysis of 366 cases (42). Sonographic criteria for early diagnosis include pseudopattern of an asymmetrical bicornuate uterus, absence of continuity between the cervical canal and horn lumen, presence of myometrium surrounding the gestational sac, and hypervascularization suggestive of placenta accreta (43). Some authors recommend 3D sonography to improve accuracy (44).

MRI was performed in 31% of cases with an accuracy of 76%. No systematic data exist on sensitivity or specificity of MRI in rudimentary horn pregnancy. Diagnostic criteria reported for MRI include a gestational sac completely surrounded by myometrium in a non-communicating horn, often located adjacent to the round ligament, with an empty primary horn displaced by the gestation (2). In developing countries, lower access to MRI led to fewer diagnoses by this modality (68% perioperative diagnoses vs. developed countries). When available, MRI accuracy did not differ between regions. Thus, while ultrasound remains the main screening tool, MRI provides valuable complementary information, improving preoperative planning and minimizing surgical emergencies.

### Management and surgical evolution

Management has advanced since the 1990s. The first laparoscopic resection was described in 1998 (29). Laparoscopic resection consists of mobilization of the horn, ligation and division of the round ligament and vessels, excision of the horn with ipsilateral salpingectomy, careful dissection and hemostasis, closure of any myometrial defect, and specimen retrieval in a containment bag. This minimally invasive approach has proven to be safe and effective for unruptured pregnancies in stable patients. Several techniques to separate the band between horns and prevent uterine rupture have been described, including staplers (29), endoscopic loop sutures (45), and vessel-sealing devices (40). Laparoscopic management has been reported up to 19 weeks of gestation (7), where the surgeon used a 4 cm hand-assist port to deliver the fetus intact. To date, none of the reported cases has used a robotic surgical system. A recent case report of robotically treated interstitial pregnancy on a tubal stump further supports the feasibility of robotic-assisted conservative surgery for rare ectopic localizations and aligns with our findings regarding precise dissection, hemostasis, and fertility preservation (46). Our findings demonstrate that robotic assistance can extend the advantages of minimally invasive surgery—enhanced precision, ergonomics, and fertility preservation—to even the most complex uterine malformations.

### Strengths and limitations of the review

A major strength of this study is its comprehensive and up-to-date systematic review, which adheres closely to PRISMA 2020 guidelines and encompasses all available published cases of non-communicating rudimentary horn pregnancies in the 21st century. The rigorous extraction and harmonization of data from a large global cohort provide insights into epidemiology, diagnosis, and management across diverse clinical settings. Additionally, the inclusion of the first case of robotic-assisted resection adds a unique perspective on minimally invasive surgical advancements.

However, several limitations must be acknowledged. The analysis relies primarily on retrospective case reports and series, which are subject to publication and reporting bias, heterogeneous diagnostic criteria, and incomplete clinical data, particularly regarding long-term reproductive outcomes and fetal prognosis. The rarity of this condition may also lead to underreporting, especially in low-resource settings where advanced imaging and documentation are limited. Comparative analyses between treatment modalities should be interpreted with caution due to the absence of randomized data and small sample sizes for certain subgroups, such as those treated robotically. Overall, this review provides the most extensive overview of rudimentary horn pregnancies to date and highlights the urgent need for standardized reporting and collaborative data collection in this rare condition.

Rudimentary horn pregnancy remains a rare but high-risk condition with significant rates of rupture and placenta accreta. Early diagnosis by advanced imaging is essential but often challenging. Minimally invasive techniques, including our first reported robotic case, demonstrate safe and effective management in selected patients. Continued systematic reporting is crucial to improve diagnosis, guide treatment strategies, and enhance patient outcomes.

## Data Availability

The original contributions presented in the study are included in the article/Supplementary Material, further inquiries can be directed to the corresponding author.
