# Supplementary material for: Robotic resection of a rudimentary horn pregnancy with the Da Vinci system: first case and systematic review of published cases in the 21st century
Source: Front Surg. 2026 May 18;13:1757761. doi: 10.3389/fsurg.2026.1757761 (PMC13223003; doi:10.3389/fsurg.2026.1757761)
Supplement: Supplementary file 1 [file Table1.docx]

| Reference | Year of publication | Country | Age | Gravida | Para | Weeks of pregnancy | hCG | Heart activity | Known/Unknown Diagnosis | Associated anomalies | Pain | Vomiting | Hypovolemic shock | Bleeding | Ultrasound | MRI | Cortison | MTX | Laparotomie | Laparoscopy | Da Vinci | Weeks of pregnancy | Rupture | Accreta | Side | Sort of anomalie: | Estimated blood loss | Tranfusion | Living infant | Remarque |
| --- | --- | --- | --- | --- | --- | --- | --- | --- | --- | --- | --- | --- | --- | --- | --- | --- | --- | --- | --- | --- | --- | --- | --- | --- | --- | --- | --- | --- | --- | --- |
| 1 | 1992 | Austria | 22 | 3 | 0 | 5 | 115 | No | Known | Agenesis of left kidney, ovary and Fallopian tube | No | No | No | No | Yes | NA |  | Yes |  |  |  |  | No | NA | right | Thick stalk | NA | NA |  | MTX in situ |
| 2 | 1997 | Japan | 26 | 1 | 0 | 10 | 8000 | NA | Unknown | anorectal agenesie, vaginal agenesie, uterus connected with colon | Yes | No | No | Yes | No | NA |  |  | Yes |  |  |  | No | NA | left | NA | NA | NA |  |  |
| 3 | 1985 | Hong-Kong | 27 | 2 | 0 | 21 |  | No | Unknown | Bigger right kidney | No | No | No | No | No | NA | No |  | Yes |  |  | 34 | No | Yes | right | NA | NA | NA |  | Preeclampsy, intrauterine desmise by anoxia |
| 4 | 2003 | USA | 24 | 2 | 0 | 6 |  | Yes | Unknown | Duplicated right ureter | No | No | No | No | No | NA |  | Yes |  |  |  |  | No | NA | right | NA | NA | No |  | Serie 11 casses |
| 5 | 2010 | Hong Kong | 33 | 2 | 1 | 5 |  | na | Unknown | na | No | No | No | yes | No | NA |  |  |  | Yes |  |  | No | NA | right | No stalk | 100 | No |  | Diagnostic with laparoscopy |
| 6 | 2009 | Spain | 16 | 1 | 0 | 27 |  | No | Unknown | NA | No | NO | No | No | No | NA | No |  | Yes |  |  | 18 | No | NA | left | Large stagger | NA | No |  |  |
| 7 | 2023 | South Africa | 29 | 2 | 1 | 24 |  | No | Unknown | NA | Yes | NA | Yes | NA | NA | NA |  |  |  |  |  | 24 | Yes |  | left | Large stagger | maximal | NA |  | bleeding out, maternal and fetal death |
| 8 | 1992 | Taiwan | 28 | 1 | 0 | 11 |  | Yes | Bicornis | NA | Yes | Yes | Yes | No | No | NA | No |  | Yes |  |  | 24 | Yes | Yes | right | Large stagger | 1500 | Yes |  | Infant deceased 2h after C Sectio |
| 9 | 2013 | India | 25 | 4 | 1 | 25 |  | Yes | Unknown | NA | Yes | NA | NA | NA | Yes | NA | No |  | Yes |  |  | 25 | Yes | NA | NA | NA | a lot | Yes |  | No news from child |
| 10 | 2022 | Turkey | 19 | 1 | 0 | 25 |  | Yes | Unknown | NA | Yes | Yes | Yes | No | No | Yes | No |  | Yes |  |  | 25 | Yes | Yes | right | Large stagger | 1000ml | Yes |  | No information if living child or not |
| 11 | 1989 | Nigeria | 25 | 5 | 4 | 26 |  | Yes | Unknown | NA | No | No | NO | No | NA | NA | No |  | Yes |  |  | 26 | No | No | left | Large stagger | minimal | No |  | Preeclampsy |
| 12 | 2007 | India | 32 | 4 | 3 | 30 |  | No | Unknown | NA | No | No | No | No | Yes | NA | No |  | Yes |  |  | 26 | No | NA | left | Large stagger | NA | No |  | Laborinduction with misoprostol and ballon without success |
| 13 | 2005 | Japan | 29 | 1 | 0 | 27 |  | Yes | Unknown | NA | Yes | No | Yes | No | Yes | NA | No |  | Yes |  |  | 27 | Yes | NA | right | Large stagger | a lot | Yes |  |  |
| 14 | 2008 | Nepal | NA | 3 | 2 | 23 |  | Yes | Unknown | NA | Yes | NA | Yes | No | No | No | No |  | Yes |  |  | 27 | Yes | Yes | left | NA | NA | NA |  | Rupture with living fetus, died at 3 days |
| 15 | 1997 | USA | NA | NA | na | 29 |  | Yes | Unknown | NA | Yes | NA | NA | NA | No | NA | NA |  | Yes |  |  | 28 | No | NA | NA | NA | NA | NA |  | twin in rudimentary horn per Sectio 28. week without removing horn, second twin in horn vaginaly 8 days later |
| 16 | 2023 | Tanzania | 29 | 2 | 1 | 28 |  | Yes | Unknown | NA | Yes | No | No | No | NO | No | Yes |  | Yes |  |  | 28 | Yes | No | left | Large stagger | NA | No |  | Living birth |
| 17 | 1994 | UK | 33 | 3 | 0 | 29 |  | Yes | Unknown | NA | Yes | NA | Yes | NA | No | NA | NA |  | Yes |  |  | 29 | Yes | Yes | right | NA | 2500 | Yes |  |  |
| 18 | 2017 | India | 31 | 2 | 1 | 29 |  | No | Unknown | NA | Yes | No | Yes | No | No | NA | No |  | Yes |  |  | 29 | Yes |  | right | Large stagger | 3500 | Yes |  |  |
| 19 | 2009 | Tunisia | 31 | 2 | 1 | 30 |  | Yes | Unknown | NA | No | no | No | no | yes | yes | yes |  | yes |  |  | 30 | Yes | NA | NA | Thin stagger | 200 | No |  | Living enfant, covered rupture, enfant liying in the abdominal cavity |
| 20 | 2023 | Iran | 12 | 1 | 0 | 31 |  | NO | Unknown | NA | Yes | No | Yes | No | No | NA | No |  | Yes |  |  | 31 | Yes | NA | left | Large stagger | 2500 | Yes |  |  |
| 21 | 2017 | Saudi Arabia | 18 | 1 | 0 | 31 |  | Yes | Unknown | NA | Yes | Yes | No | No | No | NA | No |  | Yes |  |  | 31 | Yes | No | left | NA | 350 | No |  | Living enfant |
| 22 | 2011 | India | 21 | 1 | 0 | 32 |  | Yes | Unknown | NA | No | No | No | No | No | NA | Yes |  | yes |  |  | 32 | No | No | right | Large stagger | NA | No |  | Living enfant |
| 23 | 2020 | Ethiopia | 32 | 2 | 0 | 32 |  | No | Unknown | NA | No | No | No | No | No | NA | No |  | Yes |  |  | 32 | No |  | right | NA | minimal | No |  |  |
| 24 | 2011 | USA | 21 | 2 | 1 | 30 |  | Yes | Unknown | NA | Yes | No | No | Yes | No | no | NA |  | Yes |  |  | 32 | No | NA | NA | NA |  | No |  | Living enfant |
| 25 | 2024 | Ethiopia | 38 | 2 | 1 | 32 |  | No | Unknown | NA | YEs | NA | Yes | NA | No | NA | No |  | Yes |  |  | 32 | Yes | NA | left | Large stagger | 2500 | Yes |  | Torsion and Rupture |
| 26 | 2005 | Korea | 27 | 1 | 0 | 34 |  | Yes | Unknown | NA | No | No | No | No | No | NA | No |  | Yes |  |  | 34 | No | No | right | NA | NA | No |  | IUGR, 17kg, living |
| 27 | 2024 | China | 37 | 3 | 1 | 34 |  | Yes | Unknown | NA | No | No | No | No | No | Yes | No |  | Yes |  |  | 34 | No | Yes | left | Large stagger | NA | No |  | Living enfant |
| 28 | 2009 | India | 20 | 1 | 0 | 35 |  | yes | Unknown | NA | No | No | No | No | No | NA | No |  | Yes |  |  | 35 | No | Yes | left | Large stagger | a lot | Yes |  | Living twins, preeclampsy |
| 29 | 2024 | Brazil | 39 | 4 | 1 | 26 |  | Yes | Unknown | NA | No | No | No | No | No | Yes | NA |  | Yes |  |  | 35 | No | NA | right | Large stagger | NA | No |  | Twins: one in the unicorn and one in the rudimentary |
| 30 | 2024 | Syria | 30 | 4 | 3 | 30 |  | Yes | Unknown | NA | Yes | NA | No | No | No | NA | Yes |  | Yes |  |  | 35 | Yes | Yes | left | NA | NA | Yes |  |  |
| 31 | 1988 | Pakistan | 20 | 1 | 0 | 30 |  | Yes | Unknown | NA | Yes | No | No | No | Bicornis | NA | No |  | Yes |  |  | 36 | No | Yes | left | Large stagger | NA | NA |  |  |
| 32 | 1999 | Taiwan | NA | NA | NA | 37 |  | Yes | Unknown | NA | NA | NA | NA | NA | No | NA | No |  | Yes |  |  | 37 | No | NA | NA | NA | NA | NA |  | Living child 2985g |
| 33 | 2014 | Nigeria | 32 | 2 | 2 | 28 |  | Yes | Unknown | NA | Yes | no | no | yes | no | NA | NA |  | yes |  |  | 37 | no | yes | left | Large stagger | a lot | Yes |  | C Section for transverse position |
| 34 | 2015 | China | 37 | 1 | 0 | 37 |  | Yes | Unknown | NA | No | No | No | No | No | NA | NA |  | Yes |  |  | 37 | No | Yes | right | Large stagger | 1500 | Yes |  |  |
| 35 | 2007 | India | 25 | 3 | 2 | 37 |  | Yes | Unknown | NA | No | No | No | No | No | NA | No |  | Yes |  |  | 37 | No | Yes | right | Large stagger | NA | No |  |  |
| 36 | 2015 | India | 26 | 3 | 2 | 37 |  | Yes | Unknown | NA | Yes | No | No | No | No | NA | No |  | Yes |  |  | 37 | No | YEs | right | No stagger | a lot | Yes 4 EK |  |  |
| 37 | 2003 | UK | 42 | 5 | 4 | 37 |  | No | Unknown | NA | Yes | No | No | No | No | NA | No |  | Yes |  |  | 37 | Yes | NA | left | NA | NA | No |  | Lithopaedion with perforation of the Horn into the Sigmoid |
| 38 | 2006 | UK | 34 | 1 | 0 | 37 |  | Yes | Unknown | NA | Yes | No | No | No | No | NA | No |  | Yes |  |  | 37 | Yes | Yes | right | Large stagger | 3500 | Yes |  |  |
| 39 | 2017 | France | 36 | 1 | 0 | 39 |  | No | Unknown | NA | Yes | No | No | No | NA | NA | No |  | Yes |  |  | 39 | No | No | right | NA | NA | No |  | Lithopedion at term, CT diagnosis, pregnancy symptoms for 18 years |
| 40 | 2011 | Nepal | 30 |  |  | 39 |  | Yes | Unknown | NA | No | No | No | No | NO | NA | No |  | Yes |  |  | 39 | No | NA | NA | NA | minimal | No |  |  |
| 41 | 1978 | USA | 17 | 1 | 0 | 21 |  | Yes | Unknown | NA | No | No | No | NO | No | NA | No |  | Yes |  |  | 39 | Yes | NA | left | Large stagger | 700 | NA |  | postpartum preeclampsy, living child |
| 42 | 2009 | India | 22 | 1 | 0 | 22 |  | Yes | Unknown | NA | Yes | No | No | No | No | NA | No |  | Yes |  |  | 40 | Yes | Yes | right | NA | NA | Yes |  | Rupture at 22. weeks of gestation, patient was released, Placenta attached to horn and peritoneum with abdominal pregnancy |
| 43 | 2023 | Nigeria | 35 | 3 | 2 | 41 |  | NO | Unknown | NA | No | No | No | No | No | NA | No |  | Yes |  |  | 41 | No | NA | right | Large stagger | NA | No |  | intrauterin death |
| 44 | 2007 | India | 25 | 2 | 0 | 41 |  | Yes | Unknown | NA | No | No | No | No | Yes | NA | No |  | Yes |  |  | 41 | No | NA | right | Large stagger | NA | No |  | IUGR 1,6kg, baby died 4th day |
| 45 | 2017 | Brazil | 22 | 1 | 0 | 44 |  | No | Unknown | NA | No | No | No | No | No | NA | no |  | yes |  |  | 44 | No | yes | right | Large stagger | NA |  |  | post term intra uterine death |
| 46 | 1986 | India | Na | 1 | 0 | > 41 |  | No | Unknown | NA | No | No | No | No | No | NA | No |  | Yes |  |  | > 41 | No | Na | right | Large stagger | NA | No |  | post term intra uterine death |
| 47 | 1986 | India | NA | 1 | 0 | > 41 |  | No | Unknown | NA | No | No | No | No | No | NA | No |  | Yess |  |  | > 41 | No | NA | right | Large stagger | NA | No |  | post term intra uterine death |
| 48 | 1986 | India | NA | 1 | 0 | >41 |  | No | Unknown | NA | No | No | No | No | No | NA | No |  | Yes |  |  | > 41 | No | NA | NA | NA | NA | No |  |  |
| 49 | 1970 | Malasia | 24 | 2 | 1 | > 40 |  | No | Unknown | NA | No | No | No | No | NA | NA | No |  | Yes |  |  | NA | No | NA | right | Large stagger | NA | No |  |  |
| 50 | 1919 | UK | 27 | 2 | 1 | > 40 |  | No | Unknown | NA | No | No | NO | No | NA | NA | No |  | Yes |  |  | NA | No | NA | right | Large stagger | NA | NA |  |  |
| 51 | 2009 | China | 27 | 3 | 2 | 5 |  | No | Unknown | NA | No | No | No | No | No | NA |  |  |  | Yes |  |  | No | NA | right | Thin stalk | 100 | No |  |  |
| 52 | 1994 | Taiwan | 28 | 1 | 0 | 14 |  | Yes | Unknown | NA | No | No | No | No | Yes (suspected) | NA |  |  | Yes |  |  |  | Ni | No | right | Thick stalk | NA | Na |  |  |
| 53 | 1972 | Sweden | 24 | 1 | 0 | 20 |  | NA | Unknown | NA | No | No | No | No | NA | NA |  |  | Yes |  |  |  | No | NA | left | NA | NA | NA |  |  |
| 54 | 1972 | Sweden | 27 | 1 | 0 | 14 |  | NA | Unknown | NA | No | No | No | No | NA | NA |  |  | Yes |  |  |  | No | NA | right | NA | NA | NA |  |  |
| 55 | 1947 | USA | 26 | 3 | 2 | 7 |  | NA | Unknown | NA | Yes | Yes | No | NO | NA | NA |  |  | Yes |  |  |  | No | NA | left | Thick stalk | NA | NA |  | Twins: one in the rudimentary and one in the unicornis with living birth after removing rudimentary horn |
| 56 | 1937 | UK | NA | 1 | 0 | 20 |  | NA | Unknown | NA | Yes | No | No | No | NA | NA |  |  | Yes |  |  |  | No | NA | left | Thick stalk | NA | NA |  |  |
| 57 | 1964 | Israel | 26 | 4 | 3 | 14 |  | No | Unknown | NA | No | No | No | No | NA | NA |  |  | Yes |  |  |  | No | NA | right | Thick stalk | NA | NA |  |  |
| 58 | 1959 | USA | 33 | 2 | 1 | 20 |  | No | Unknown | NA | No | No | No | No | NA | NA |  |  | Yes |  |  |  | No | NA | right | Thick stalk | NA | NA |  |  |
| 59 | 1944 | India | 27 | 1 | 0 | 16 |  | NA | Unknown | NA | Yes | No | NO | No | NA | NA |  |  | Yes |  |  |  | No | NA | right | Thick stalk | NA | NA |  |  |
| 60 | 1972 | Sweden | 31 | 2 | 1 | 16 |  | NA | Known | NA | No | No | No | Yes | NA | NA |  |  | Yes |  |  |  | No | NA | left | Thin stalk | NA | NA |  |  |
| 61 | 2000 | India | 18 | 1 | 0 | 14 |  | NA | Unknown | NA | Yes | No | No | No | No | NA |  |  | Yes |  |  |  | No | NA | left | NA | NA | No |  | Resection of the pregnancy only due to lack of consent to do an hysterectomie |
| 62 | 2018 | Italy | 18 | 1 | 0 | 5 | 5056 | no | Unknown | na | Yes | No | No | yes | Yes | NA |  |  |  | Yes |  |  | No | NA | right | Thin stalk | minimal | No |  |  |
| 63 | 2018 | India | 24 | 1 | 0 | 5 |  | No | Unknown | NA | No | No | No | No | Yes | NA |  |  |  | Yes |  |  | No | NA | right | Thin stalk | minimal | No |  |  |
| 64 | 2011 | Nepal | 21 | 1 | 0 | 8 |  | no | Unknown | NA | yes | no | no | yes | no | NA |  |  | yes |  |  |  | no | NA | NA | NA | minimal | no |  |  |
| 65 | 2016 | USA | 31 | 1 | 0 | 5 | 657 | No | Unknown | NA | Yes | Yes | No | No | No | Yes |  | Yes |  | Yes |  |  | No | NA | right | Thin stalk | minimal | No |  |  |
| 66 | 2019 | Portugal | 34 | 4 | 1 | 6 | 58000 | Yes | Unknown | NA | No | No | No | No | No | NA |  | Yes |  | Yes |  |  | No | NA | right | Thick stalk | minimal | No |  |  |
| 67 | 2021 | Germany | 23 | 1 | 0 | 6 | 47000 | Yes | Unknown | NA | Yes | No | No | No | Yes | NA |  |  |  | Yes |  |  | No | NA | right | Thick stalk | minimal | No |  |  |
| 68 | 2011 | Nepal | 23 | 2 | 1 | 14 |  | no | Unknown | NA | Yes | no | no | no | no | NA |  |  | yes |  |  |  | no | NA | right | NA | minimal | No |  |  |
| 69 | 2022 | China | 39 | 1 | 0 | 6 |  | NA | Known | NA | No | No | No | No | Yes | Yes |  |  |  | Yes |  |  | No | NA | right | NA | 10 | No |  |  |
| 70 | 2025 | USA | 34 | 2 | 1 | 6 |  | NA | Unknown | NA | No | No | Ni | No | Yes | Yes |  |  |  | Yes |  |  | No | NA | right | Thick stalk | minimal | No |  |  |
| 71 | 1988 | Sweden | 21 | 2 | 0 | 17 |  | Yes | Unknown | NA | No | NO | No | NO | No | NA |  |  | Yes |  |  |  | No | NA | right | Thick stalk | NA | Na |  |  |
| 72 | 2023 | Sudan | 25 | 3 | 2 | 18 |  | Yes | Unknown | NA | No | No | No | No | No | NA |  |  | Yes |  |  |  | No | NA | right | Thin stalk | minimal | No |  |  |
| 73 | 2006 | Turkey | 28 | 1 | 0 | 6 |  | Yes | Known | No | No | No | No | No | Yes | NA |  |  |  | Yes |  |  | No | NA | right | Thick stalk | NA | No |  |  |
| 74 | 2002 | USA | 21 | 1 | 0 | 6 |  | Yes | Known | No | No | No | No | No | Yes | NA |  |  |  | Yes |  |  | No | Yes | right | Thick stalk | minimal | No |  |  |
| 75 | 2015 | USA | 28 | 1 | 0 | 6 |  | Yes | Known | No | No | No | No | No | Yes | No |  |  |  | Yes |  |  | No | NA | right | NA | minimal | No |  |  |
| 76 | 1995 | Japan | 19 | 1 | 0 | 16 |  | No | Unknown | NA | No | No | No | No | No | NA |  |  | Yes |  |  |  | No | No | right | Thin stalk | minimal | No |  | Death, attempt to induce labor, C Section |
| 77 | 2010 | USA | 30 |  |  | 17 |  | No | Unknown | NA | NO | No | No | No | No | NA |  |  | Yes |  |  |  | No | Yes | NA | NA | NA | NA |  | misoprostol induction by fetal demise |
| 78 | 2022 | France | 35 | 1 | 0 | 6 |  | NA | Unknown | No | No | No | No | No | No | Yes |  |  |  | Yes |  |  | No | NA | right | Thick stalk | NA | NA |  |  |
| 79 | 2024 | UK | 29 | 2 | 0 | 6 | 28,853 | Yes | Known | No right kidney | Yes | No | No | No | Yes | Yes |  |  |  | Yes |  |  | No | NA | right | No stalk | minimal | No |  |  |
| 80 | 2007 | Turkey | 32 | 4 | 1 | 6 | 3800 | Yes | Unknown | NA | No | No | No | No | Yes | NA |  | Yes |  |  |  |  | No | NA | left | NA | NA | No |  | MTX in situ |
| 81 | 2013 | China | 27 | 3 | 0 | 9 |  | NA | Unknown | na | No | No | No | No | Yes | NA |  |  | Yes |  |  |  | No | NA | NA | NA | 20 | NA |  | Serie 11 casses |
| 82 | 1998 | Japan | 22 | NA | NA | 7 |  | NA | NA | NA | NA | NA | NA | NA | Yes | NA |  |  | Yes |  |  |  | No | NA | NA | NA | NA | NA |  |  |
| 83 | 2018 | USA | 31 | 3 | 1 | 8 | 123500 | Yes | known | NA | No | no | No | no | yes | na |  | yes | yes |  |  |  | No | na | right | na | minimal | No |  |  |
| 84 | 2022 | Japan | 30 | 1 | 0 | 6 | 25000 | Yes | Unknown | No right kidney | No | No | No | No | Yes | Yes |  | No |  | Yes |  |  | No | Yes | right | No stalk | minimal | No |  |  |
| 85 | 1998 | Japan | 22 | NA | NA | 7 |  | Yes | Unknown | NA | NA | NA | NA | NA | Yes | NA |  |  |  | Yes |  |  | NA | NA | NA | NA | NA | NA |  |  |
| 86 | 2008 | India | 20 | 1 | 0 | 11 |  | No | Unknown | NA | No | No | No | No | Yes | NA |  |  | Yes |  |  |  | No | NA | left | Thick stalk | NA | No |  |  |
| 87 | 2003 | China | 29 | 3 | 1 | 7 |  | NA | Unknown | na | No | No | No | No | No | na |  | MTX |  | Yes |  |  | No | NA | right | NA | 100 | NA |  | Serie 11 casses |
| 88 | 2024 | USA | 30 | 5 | 1 | 7 | 22500 | Yes | Known | NA | No | No | No | No | Yes | NA |  |  |  | Yes |  |  | No | No | right | Thin stalk | 20 | No |  |  |
| 89 | 2022 | Taiwan | 37 | 2 | 1 | 7 | 20000 | NA | Unknown | NA | Yes | No | No | No | No | NA |  | Yes |  | Yes |  |  | No | NA | right | Thick stalk | minimal | No |  | CT diagnosis of ectopic pregnancy, resection only the pregnancy |
| 90 | 2008 | France | 28 | 2 | 0 | 7 | 10091 | Yes | Unknown | No | No | No | No | No | Yes | NA |  |  |  | Yes |  |  | No | Yes | left | Thick stalk | NA | No |  |  |
| 91 | 2015 | China | 26 | 1 | 0 | 7 |  | Yes | Unknown | No | Yes | No | No | No | Yes | No |  |  |  | Yes |  |  | No | NA | left | Thin stalk | minimal | No |  |  |
| 92 | 2022 | France | 26 | 1 | 0 | 7 |  | NA | Unknown | No | No | No | No | No | No | Yes |  |  |  | Yes |  |  | No | NA | left | NA | NA | NA |  |  |
| 93 | 2024 | China | 27 | 1 | 0 | 7 | > 200 000 | Yes | Unknown | No | No | No | NO | No | Yes | Yes |  |  |  | Yes |  |  | No | NA | left | Thick stalk | minimal | No |  |  |
| 94 | 2021 | UK | 27 | 1 | 0 | 12 |  | Yes | Unknown | NA | Yes | No | No | No | Yes (suspected) | NA |  |  | Yes |  |  |  | No | NA | right |  | minimal | No |  |  |
| 95 | 2017 | China | 29 | 3 | 1 | 7 |  | No | Known | no | no | no | no | no | yes | yes |  | yes |  | yes |  |  | No | NA | right | Thick stalk | minimal | No |  | Invasive Mole in rudimentary Horn |
| 96 | 2016 | India | 29 | 1 | 0 | 19 |  | Yes | Bicornis | NA | Yes | No | No | No | No | No |  |  | Yes |  |  |  | No | No | left | NA | 300 | No |  | Suspicion of rupture |
| 97 | 2002 | Belgium | 23 | 1 | 0 | 7 |  | NA | Known | NA | No | No | No | No | No | Yes |  |  | Yes |  |  |  | No | NA | left | NA | minimal | No |  |  |
| 98 | 2019 | Germany | 16 | 1 | 0 | 7 | 61000 | NA | Unknown | None | No | Yes | No | No | No | NA |  |  |  | Yes |  |  | No | NA | right | No stalk | minimal | No |  | induction with misoprostol without success |
| 99 | 2012 | Turkey |  |  |  |  |  |  | Unknown | NA | NA | NA | NA | NA | No | Yes |  |  |  |  |  |  | No | NA | NA | NA | NA | No |  | Diagnosis with saline infusion |
| 100 | 1998 | USA | 34 | 1 | 0 | 14 |  | Yes | Unknown | NA | Yes | No | No | No | No | Yes |  |  | Yes |  |  |  | No | NA | NA | NA | NA | NA |  |  |
| 101 | 2014 | China | 32 | 1 | 0 | 8 |  | NA | Unknown | NA | No | No | No | No | No | NA |  |  |  | Yes |  |  | No | NA | NA | NA | 5 | NA |  | Serie 11 casses |
| 102 | 2005 | Israel | 34 | 5 | 4 | 11 | 30000 | No | Bicornis | NA | No | No | No | No | No | Yes |  |  | Yes |  |  |  | No | Yes | left | NA | NA | No |  |  |
| 103 | 1999 | China | 26 | 1 | 0 | 8 |  | NA | Unknown | NA | No | No | No | No | No | NA |  |  |  | Yes |  |  | No | NA | right | NA | 10 | NA |  | Perforation of left horn with an aspiration to terminate pregnancy |
| 104 | 2014 | Turkey | 37 | 3 | 2 | 8 | 30782 | Yes | Unknown | na | Yes | no | no | no | no | NA |  |  |  | Yes |  |  | No | NA | right | Thin stalk | minimal | No |  |  |
| 105 | 1998 | Israel | 31 | 2 | 1 | 8 | 29000 | Yes | Unknown | NA | Yes | No | No | No | Yes | NA |  |  |  | Yes |  |  | No | NA | right | NA | minimal | No |  |  |
| 106 | 2005 | Israel | 26 | 1 | 0 | 8 |  | Yes | Unknown | NA | No | No | No | No | No | Yes |  |  | Yes | Yes |  |  | No | Yes | right | Thick stalk | NA | No |  | 13. week for surgery |
| 107 | 2010 | The Netherlands | 33 | 3 | 1 | 9 |  | NA | Unknown | NA | No | NO | No | No | Yes | Yes |  |  | Yes |  |  |  | No | NA | right | Thick stalk | NA | No |  | Diag Hysteroskopie to make the diagnosis by suspected bicornis |
| 108 | 2024 | Argentina | 38 | 11 | 6 | 8 | 150000 | Yes | Known | NA | no | No | No | No | Yes | Yes |  |  |  | Yes | s |  | No | NA | right | Thin stalk | minimal | No |  |  |
| 109 | 1995 | India | 22 | 1 | 0 | 8 |  | Yes | Unknown | No | No | No | No | No | Yes (suspected) | NA |  |  | Yes | Yes |  |  | No | NA | right | Thick stalk | NA | No |  | Curetage before laparotomy to confirm rudimentary horn |
| 110 | 2025 | Marocco | 31 | 2 | 0 | 8 |  | Yes | Unknown | No | Yes | No | No | No | Yes | Yes |  |  |  | Yes |  |  | No | NA | left | Thick stalk | minimal | No |  |  |
| 111 | 2007 | USA | 36 | 2 | 1 | 8 | 89000 | Yes | Known | No | No | No | No | No | Yes | Yes |  | Yes |  | Yes |  |  | No | NA | right | Thick stalk | 50 | No |  | MTX and KCl in the pregnancy and then Resection by hCG 600 |
| 112 | 2022 | Canada | 27 | 3 | 0 | 18 |  | Yes | Unknown | NA | No | No | No | No | Yes | Yes |  |  | Yes |  |  |  | No | Yes | left | Thick stalk | minimal | No |  | Inccreta |
| 113 | 2018 | India | 16 | 1 | 0 | 17 |  | no | Unknown | NA | Yes | no | no | no | no | NA |  |  | yes |  |  |  | yes | NA | right | Thin stalk | na | Yes |  | Torsion of the Horn, temptation of vaginal deliverie for feath fetus |
| 114 | 1961 | UK | 22 | 1 | 0 | 17 |  | NA | Unknown | NA | Yes | Yes | Yes | No | NA | NA |  |  | Yes |  |  |  | Yes | NA | left | NA | 3000 | Yes |  |  |
| 115 | 1854 | Deutschland | 28 | 5 | 3 | 20 |  | NA | Unknown | NA | Yes | No | Yes | No | NA | NA |  |  | No |  |  |  | Yes | NA | left | NA | maximal | NA |  | Death report |
| 116 | 1992 | USA | NA | NA | NA | 19 |  | NA | Unknown | NA | Yes | NA | Yes | NA | NA | NA |  |  | Yes |  |  |  | yes | NA | right | NA | NA | NA |  | Twin pregnancy, one in rudimentary horn, second in unicornate horn born 1 day later |
| 117 | 1909 | UK | 19 | 1 | 0 | 20 |  | NA | Unknown | NA | Yes | NA | Yes | No | NA | NA |  |  | No |  |  |  | Yes | NA | right | NA | maximal | NA |  | died 15 min after admission |
| 118 | 1955 | Iran | 19 | 3 | 1 | 20 |  | No | Unknown | NA | Yes | Yes | Yes | No | NA | NA |  |  | Yes |  |  |  | Yes | NA | left | Thick stalk | NA | Yes |  |  |
| 119 | 2018 | Tunesia | 35 | 1 | 0 | 16 |  | No | Unknown | NA | NA | NA | Yes | NA | NA | NA |  |  |  |  |  |  | Yes | NA | right | Thick stalk | maximal | No |  | Death, autopsie of the mother |
| 120 | 1993 | Thailand | 37 | 2 | 1 | 15 |  | No | Unknown | NA | Yes | No | Yed | No | NA | NA |  |  | Yes |  |  |  | Yes | NA | right | Thick stalk | 4000 | NA |  |  |
| 121 | 1959 | Israel | 18 | 1 | 0 | 18 |  | NA | Unknown | NA | Yes | Yes | Yes | Yes | NA | NA |  |  | Yes |  |  |  | Yes | NA | left | Thin stalk | a lot | NA |  |  |
| 122 | 1874 | Deutschland | 27 | 2 | 1 | 20 |  | NA | Unknown | NA | Yes | NA | Yes | No | NA | NA |  |  | No |  |  |  | Yes | NA | left | Thin stalk | maximal | NA |  | Death report |
| 123 | 1952 | USA | 25 | 1 | 0 | 12 |  | NA | Unknown | NA | Yes | yes | Yed | No | NA | NA |  |  | yes |  |  |  | yes | NA | right | Thin stalk | NA | Yes |  |  |
| 124 | 2004 | China | 31 | NA | NA | 12 |  | No | Unknown | NA | Yes | NA | Yes | No | NA | NA |  |  | Yes |  |  |  | Yes | No | left | NA | NA | NA |  |  |
| 125 | 2000 | China | 25 | 1 | 0 | 9 |  | NA | Unknown | NA | Yes | NA | Yes | yes | No | NA |  |  | Yes | Yes |  |  | No | NA | NA | NA | 200 | NA |  |  |
| 126 | 2013 | UK | 38 | 8 | 5 | 19 |  | yes | Unknown | NA | Yes | NA | yes | no | no | NA |  |  | yes |  |  |  | yes | Na | left | NA | a lot | Yes |  |  |
| 127 | 2025 | USA | 30 | 3 | 1 | 9 |  | Yes | Unknown | NA | No | No | No | No | Yes | Yes |  |  |  | Yes |  |  | No | NA | right | Thin stalk | minimal | No |  |  |
| 128 | 2005 | USA | NA | 1 | 0 | 18 |  | Yes | Bicornis | NA | NA | NA | NA | NA | No | NA |  |  | Yes |  |  |  | Yes | NA | left | NA | NA | NA |  |  |
| 129 | 2005 | UK | 39 | 2 | 1 | 16 |  | No | Unknown | NA | Yes | No | Yes | No | No | NA |  |  | Yes |  |  |  | Yes | NA | left | NA | 3500 | Yes |  |  |
| 130 | 2004 | UK | 23 | 1 | 0 | 15 |  | No | Unknown | NA | Yes | NA | Yes | No | No | NA |  |  | Yes |  |  |  | Yes | NA | left | NA | 3000 | Yes |  | rudimentary Horn left in place and repared |
| 131 | 1998 | UK | NA | 1 | 0 | 9 |  | Yes | Unknown | No | Yes | No | No | Yes | No | NA |  |  |  | Yes |  |  | No | NA | left | Thick stalk | NA | No |  | MTX im and intraamnial, then resection with laparoscopy |
| 132 | 2000 | China | 26 | 2 | 0 | 11 |  | NA | Unknown | NA | Yes | Yes | Yes | No | No | NA |  |  | Yes |  |  |  | Yes | NA | NA | Na | 2500 | NA |  | Serie 11 casses |
| 133 | 1993 | China | 25 | 1 | 0 | 12 |  | NA | Unknown | na | Yes | Yes | Yes | No | No | NA |  |  | Yes |  |  |  | Yes | NA | NA | NA | 2500 | NA |  | Serie 11 casses |
| 134 | 2013 | China | 25 | 1 | 0 | 14 |  | NA | Unknown | NA | Yes | NA | Yes | No | No | NA |  |  | Yes |  |  |  | Yes | NA | NA | NA | 3000 | NA |  | Serie 11 casses |
| 135 | 2011 | China | 26 | 3 | 1 | 22 |  | NA | Unknown | NA | Yes | NA | Yes | No | No | NA |  |  | Yes |  |  |  | Yes | NA | NA | NA | 2500 | NA |  | Serie 11 casses |
| 136 | 1986 | India | NA | 2 | 1 | 13 |  | NA | Unknown | NA | Yes | NA | Yes | NA | No | NA |  |  | yes |  |  |  | Yes | NA | NA | NA | 2500 | Yes |  |  |
| 137 | 1986 | India | NA | 1 | 0 | 15 |  | NA | Unknown | NA | Yes | NA | Yes | NA | No | NA |  |  | yes |  |  |  | Yes | NA | NA | NA | 2000 | Yes |  |  |
| 138 | 2012 | Singapore | 23 |  |  | 10 |  | Yes | Unknown | NA | No | No | No | no | No | NA |  |  |  | yes |  |  | No | No | left | Thin stalk | minimal | No |  | Diagnostic with hysteroscopy |
| 139 | 2024 | India | 23 | 1 | 0 | 10 |  | NA | Unknown | NA | No | No | No | No | Yes | NA |  |  |  | Yes |  |  | No | NA | right | Thin stalk | minimal | No |  |  |
| 140 | 2013 | India | 30 | 2 | 1 | 10 |  | No | Unknown | na | Yes | yes | yes | yes | no | NA |  |  | yes |  |  |  | yes | NA | right | NA | NA | no |  | False diagnosis of iliac externa aneuvrysma |
| 141 | 2013 | Denmark |  | 1 | 0 | 21 |  | yes | Unknown | NA | Yes | no | yes | no | No | NA |  |  | yes |  |  |  | yes | NA | right | NA | 5000 | NA |  | BMI 36 |
| 142 | 2013 | Japan | 30 | 1 | 0 | 19 |  | No | Unknown | NA | Yes | NA | Yes | NA | No | NA |  |  | yes |  |  |  | yes | NA | right | NA | maximal | No |  | Death, autopsie of the mother, 3 days after first pain |
| 143 | 2024 | Nepal | 25 | 1 | 0 | 16 |  | No | Unknown | NA | Yes | No | Yes | No | No | NA |  |  | Yes |  |  |  | Yes | NA | left | Thick stalk | 3500 | Yes |  |  |
| 144 | 2022 | Dublin | 20 | 1 | 0 | 10 |  | Yes | Unknown | NA | No | No | No | No | Yes | Yes |  |  |  | Yes |  |  | No | Yes | left | Thick stalk | minimal | No |  |  |
| 145 | 2009 | Nigeria | 26 | 3 | 0 | 20 |  | Yes | Unknown | NA | Yes | Yes | Yes | No | No | NA |  |  | Yes |  |  |  | Yes | NA | left | Thick stalk | 1500 | Yes |  |  |
| 146 | 2025 | Malaysia | 20 | 1 | 0 | 21 |  | Yes | Unknown | NA | Yes | No | Yes | No | No | NA |  |  | Yes |  |  |  | Yes | NA | right | Thick stalk | 1000 | NA |  |  |
| 147 | 2021 | Morocco | 32 | 3 | 2 | 16 |  | No | Unknown | NA | Yes | No | Yes | No | No | NA |  |  | Yes |  |  |  | Yes | NA | right | Thick stalk | 3000 | Yes |  | Explorative Laparotomie, Hb 4 |
| 148 | 2020 | Nepal | 25 | 3 | 2 | 15 |  | No | Unknown | NA | Yes | Yes | Yes | No | No | NA |  |  | Yes |  |  |  | Yes | NA | right | Thick stalk | 1500 | Yes |  | Explorative Laparotomie, Hb 2,8 |
| 149 | 2013 | India | 24 | 1 | 0 | 10 |  | NA | Unknown | NA | Yes | No | Yes | No | No | NA |  |  |  | yes |  |  | Yes | NA | left | NA | 2500 | yes |  |  |
| 150 | 2014 | Cameroun | 22 | 2 | 1 | 17 |  | No | Unknown | NA | Yes | YEs | Yes | No | No | NA |  |  | yes |  |  |  | Yes | NA | right | Thick stalk | 3500 | Yes |  |  |
| 151 | 2020 | Egypte | 25 | 2 | 1 | 20 |  | No | Unknown | NA | Yes | No | Yes | No | No | NA |  |  | Yes |  |  |  | Yes | No | right | Thick stalk | NA | Yes |  | Triplet induced with Clomid, cerclage |
| 152 | 2010 | Kosovo | 22 | 2 | 1 | 16 |  | NA | Unknown | NA | Yes | No | Yes | No | No | NA |  |  | Yes |  |  |  | Yes | No | right | Thin stalk | 1500 | Yes |  |  |
| 153 | 2011 | India | 26 | 3 | 1 | 16 |  | No | Unknown | NA | Yes | No | Yes | Yes | No | NA |  |  | Yes |  |  |  | Yes | No | right | Thin stalk | maximal | Yes |  |  |
| 154 | 1983 | China | 31 | 3 | 1 | 23 |  | No | Unknown | NA | Yes | NA | Yes | No | No | NA |  |  | Yes |  |  |  | Yes | yes | NA | NA | 2000 | NA |  | Serie 11 casses |
| 155 | 2003 | China | 24 | 1 | 0 | 10 |  | NA | Unknown | NA | No | NA | No | yes | No | NA |  |  | Yes | Yes |  |  | Yes | NA | NA | NA | 100 | NA |  | Serie 11 casses |
| 156 | 2022 | China | 35 | 4 | 0 | 17 |  | Yes | Unknown | NA | Yes | No | Yes | No | No | NA |  |  | Yes |  |  |  | Yes | Yes | left | Thick stalk | 500ml | Yes |  | CT diagnosis of rupture |
| 157 | 2011 | Japan | 27 | 2 | 1 | 20 |  | Yes | Unknown | NA | yes | No | No | No | Yes | NA |  |  | Yes |  |  |  | Yes | NA | right | NA | 3300 | yes |  | Covered rupture with small intestine, infection of the uterine horn |
| 158 | 2018 | Iran | 24 | 1 | 0 | 12 |  | Yes | Unknown | NA | Yes | Yes | Yes | No | yes | NA |  |  | Yes |  |  |  | Yes | NA | left | Thick stalk | 1000 | No |  | Living twin pregnancy, C Sectio 38. week |
| 159 | 2012 | India | 26 | 2 | 1 | 18 |  | No | Unknown | na | Yes | yes | yes | no | yes | NA |  |  | yes |  |  |  | yes | no | left | Thick stalk | 4000 | Yes |  |  |
| 160 | 2010 | Denmark | 34 | 4 | 3 | 16 | 22252 | No | Unknown | NA | Yes | No | Yes | No | No | No |  |  | Yes |  |  |  | Yes | NA | left | NA | 4800 | Yes |  |  |
| 161 | 2015 | Turkey | 29 | 3 | 2 | 16 |  | Yes | Unknown | NA | Yes | Yes | Yes | no | no | no |  |  | Yes |  |  |  | Yes | NA | right | NA | 3000 | NA |  |  |
| 162 | 2015 | China | 27 | 1 | 0 | 15 |  | No | Unknown | NA | Yes | No | Yes | No | Yes | No |  |  | Yes |  |  |  | Yes | NA | left | Thick stalk | 1500 | NA |  |  |
| 163 | 2024 | Saudi Arabian | 39 | 4 | 3 | 19 |  | Yes | Unknown | NA | Yes | No | Yes | No | Yes | Yes |  |  | Yes |  |  |  | Yes | NA | left | Thick stalk | 1000 | Yes |  |  |
| 164 | 2010 | USA | 38 | 3 | 2 | 21 |  | Yes | Unknown | NA | No | No | No | No | Yes | YEs |  |  | Yes |  |  |  | Yes | No | right | Thick stalk | NA | No |  | Arm, umbilical cord hernied in the main uterin cavity, C Section at 24+3, deformation and edema of the lower face, no airway possible |
| 165 | 2022 | France | 37 | 3 | 1 | 10 |  | NA | Unknown | No | No | No | No | No | Yes | Yes |  |  |  | Yes |  |  | No | NA | NA | NA | NA | NA |  |  |
| 166 | 2005 | India | 19 | 1 | 0 | 16 |  | NA | Unknown | NA | Yes | NA | Yes | No | No | NA |  |  | Yes | No |  |  | Yes | No | left | NA | NA | NA |  |  |
| 167 | 2012 | India | 25 | 2 | 1 | 25 |  | No | Unknown | No | Yes | No | Yes | No | No | NA | No |  | Yes |  |  | 25 | Yes | NA | right | Large stagger | 3000 | Yes |  | Misoprostol induction for intrauterine fetal desmise |
| 168 | 2022 | Turkey | 19 | 2 | 0 | 25 |  | Yes | Unknown | No | Yes | Yes | Yes | No | No | Yes | NA |  | Yes |  |  | 25 | Yes | Yes | right | Large stagger | 1000 | Yes |  | Living birth when delivered |
| 169 | 2003 | Japan | 29 | 1 | 0 | 26 |  | Yes | Unknown | No | Yes | No | No | No | No | NA | No |  | Yes |  |  | 26 | Yes | Yes | left | NA | 2000 | Yes |  |  |
| 170 | 1988 | Finland | 24 | 3 | 1 | 17 |  | Yes | Known | No | Yes | No | No | No | No | NA | No |  | Yes |  |  | 30 | Yes | Yes | right | Large stagger | 1500 | Yes |  |  |
| 171 | 1983 | Denmark | 29 | 1 | 0 | 31 |  | Yes | Unknown | No | Yes | No | No | No | No | NA | No |  | Yes |  |  | 31 | Yes | yes | right | Large stagger | 200 | NA |  | living infant |
| 172 | 2015 | Oman | 31 | 5 | 2 | 32 |  | Yes | Unknown | No | Yes | no | no | no | No | Yes | NA |  | Yes |  |  | 32 | Yes | Yes | left | Thin stagger | 2300 | Yes |  |  |
| 173 | 1998 | UK | 24 | 1 | 0 | 14 |  | Yes | Unknown | No | Yes | No | Yes | Yes | No | NA | Yes |  | Yes |  |  | 34 | Yes | NA | left | Large stagger | 3000 | Yes |  | intrauterin death |
| 174 | 2016 | India | 23 | 2 | 0 | 34 |  | Yes | Unknown | No | No | No | No | No | No | NA | No |  | Yes |  |  | 34 | Yes |  | right | Large stagger | a lot | Yes, 6 EK |  | Rupture of the Horn during pregnancy, Pregnancy continued abdominal with adhesions to sigmoid colon |
| 175 | 2009 | Turkey | 27 | 1 | 0 | 37 |  | Yes | Unknown | No | No | No | No | No | No | NA | No |  | Yes |  |  | 37 | No | NA | right | Large stagger |  | no |  | Misdiagnose of bironuate uterus in an early laparoscopy as well |
| 176 | 2011 | India | 24 | 3 | 1 | 34 |  | Yes | Unknown | No | Yes | No | No | No | No | NA | No |  | Y |  |  | 37 | Yes | yes | right | Large stagger | 2500 | Yes |  | Baby well, abdominal pregnancy, Placenta attached to the Horn |
| 177 | 1951 | UK | 25 | 2 | 0 | 41 |  | NA | Unknown | No | No | No | No | No | NA | NA | No |  | Yes |  |  | 41 | No | No | right | Large stagger | NA | Na |  | living child 2720g |
| 178 | 2016 | Cameroun | 29 | 2 | 0 | 42 |  | No | Unknown | No | NO | No | No | No | No | NA | No |  | Yes |  |  | 42 | No | NA | right | Large stagger | minimal | No |  |  |
| 179 | 2008 | Israel | 37 | 1 | 0 | 11 |  | No | Unknown | NA | Yes | No | No | Yes | Yes | NA |  | No |  | Yes |  |  | No | NA | left | Thick stalk | NA | No |  |  |
| 180 | 1994 | Japan | 23 | 1 | 0 | 16 |  | No | Unknown | No | Yes | No | No | No | No | NA |  |  | Yes |  |  |  | No | NA | right | NA | minimal | No |  | Diag Hysteroskopie to make the diagnosis |
| 181 | 2008 | Ghana | 28 | 6 | 5 | 16 |  | No | Unknown | No | No | No | No | Yes | No | NA |  |  | Yes |  |  |  | No | NA | left | Thick stalk | 200 | No |  |  |
| 182 | 2004 | UK | 32 | NA | NA | 12 | 71000 | Yes | Unknown | NA | No | No | No | No | Yes | NA |  | Yes |  | Yes |  |  | No | NA | right | Thick stalk | NA | No |  | Surgery after 6 Months (KCl intraamnial and MTX IM) |
| 183 | 2011 | India | 33 | 3 | 1 | 12 |  | Yes | Unknown | NA | No | No | No | NO | Yes | Yes |  |  |  | Yes |  |  | No | NA | right | Thick stalk | minimal | No |  | Horn retrieved through posterior colpotomy |
| 184 | 2003 | USA | 24 | 3 | 2 | 16 |  | Yes | Unknown | No | Yes | No | No | No | No | NA |  |  | Yes |  |  |  | No | NA | right | Thin stalk | NA | No |  |  |
| 185 | 1979 | Sweden | 27 | 3 | 0 | 22 |  | No | Unknown | No | No | No | No | Yes | No | NA |  |  | Yes |  |  |  | No | NA | right | Thin stalk | NA | NA |  |  |
| 186 | 2020 | France | 18 | 2 | 0 | 12 |  | Yes | Unknown | NA | Yes | Yes | No | No | No | NA |  |  | Yes | Yes |  |  | Yes | Na | right | Thick stalk | 1000 | Yes |  | explorative Laparoskopy and then Laparotomie |
| 187 | 2024 | USA | 25 | 3 | 2 | 12 |  | Yes | Unknown | No | No | No | No | Ni | No | NA |  |  |  | Yes |  |  | No | NA | left | Thin stalk | minimal | No |  |  |
| 188 | 1998 | Korea | 23 | 1 | 0 | 8 |  | Yes | Unknown | No | No | No | No | No | Yes | NA |  |  | Yes |  |  |  | No | NA | right | Thick stalk | minimal | No |  |  |
| 189 | 2013 | Canada | 28 | 1 | 0 | 12 |  | yes | Unknown | no | no | no | no | no | yes | yes |  |  |  | yes |  |  | no | no | left | Thick stalk | minimal | no |  | fetal intracardiac KCl |
| 190 | 2012 | Tunesia | 32 | 1 | 0 | 12 |  | no | Unknown | No | No | No | No | no | Yes (suspected) | Yes |  |  | Yes | yes |  |  | No | NA | left | Thin stalk | minimal | no |  |  |
| 191 | 1998 | Canada | 34 | 2 | 1 | 10 |  | Yes | Unknown | No | Yes | No | No | No | Yes | NA |  |  | Yes |  |  |  | No | Yes | right | Thick stalk | NA | No |  | Termination at 17th week of twin in rudimentary horn, C Sectio 36. week and exzicion of rudimentary horn |
| 192 | 2018 | USA | 34 | 1 | 0 | 12 |  | Yes | Unknown | no | Yes | No | No | No | No | Yes |  |  | Yes | Yes |  |  | Yes | NA | left | NA | moderate | No |  | Twins, Rupture at 17. weeks, Termination at 19. weeks because of severe heart anoamlie for fetus 2 |
| 193 | 2022 | France | 22 | 5 | 2 | 12 |  | NA | Unknown | No | No | No | No | No | No | No |  |  |  | NA |  |  | No | NA | NA | NA | NA | NA |  |  |
| 194 | 2022 | France | 27 | 1 | 0 | 13 |  | NA | Unknown | No | No | No | No | No | No | Yes |  |  | Yes | Yes |  |  | No | NA | right | Thick stalk | NA | NA |  |  |
| 195 | 2013 | India | 21 | 1 | 0 | 14 |  | 2 | Unknown | NA | No | No | No | No | Yes | NA |  |  |  | Yes |  |  | No | NA | left | NA | NA | No |  |  |
| 196 | 2013 | India | 24 | 5 | 1 | 14 |  | NA | Unknown | NA | No | No | No | No | Yes | NA |  |  |  | Yes |  |  | No | NA | left | Thin stalk | NA | Yes |  | SeHemorrhage on day 1 postoperatively from left round ligament stump with multiple blood transfusion |
| 197 | 2020 | Swizerland | 36 | 2 | 0 | 15 |  | Yes | Unknown | NA | Yes | No | No | No | No | NA |  |  |  | Yes |  |  | Yes | NA | left | Thick stalk | 2000 | Yes |  | Explorative Laaproscopy |
| 198 | 2008 | UK | 23 | 2 | 1 | 15 |  | Yes | Unknown | NA | Yes | Yes | Yes | Yes | no | NA |  |  | Yes | Yes |  |  | Yes | Yes | Na | Stalk | 5000 | Yes |  | Twin pregnancy with one acardiac |
| 199 | 2022 | France | 16 | 1 | 0 | 12 |  | NA | Unknown | No | Yes | No | No | Yes | No | Yes |  |  |  | NA |  |  | No | NA | right | Thick stalk | NA | NA |  |  |
| 200 | 2017 | Egypte | 16 | NA | NA | 16 |  | No | Unknown | NA | No | No | No | No | No | NA |  |  | yes | yes |  |  | No | NA | right | NA | NA | No |  | Misoprostol and curetage with perforation and urgency laparotomie |
| 201 | 2004 | Turkey | 26 | 1 | 0 | 18 |  | Yes | Unknown | No | Yes | No | No | Yes | No | Yes |  |  | Yes |  |  |  | No | NA | right | Thick stalk | NA | No |  |  |
| 202 | 2022 | France | 27 | 1 | 0 | 18 |  | NA | Unknown | No | No | No | No | No | No | Yes |  |  | Yes |  |  |  | No | NA | NA | Thin stalk | NA | NA |  |  |
| 203 | 2015 | Canada | 27 | NA | NA | 16 |  | NA | NA | NA | NA | NA | NA | NA | NA | No |  | Yes |  | Yes |  |  | No | NA | left | NA | NA | NA |  |  |
| 204 | 2006 | Canada | 31 | 1 | 0 | 13 |  | Yes | Unknown | No | No | No | No | No | Yes | Yes |  |  | Yes |  |  |  | No | NA | right | NA | NA | no |  |  |
| 205 | 2022 | USA | 21 | 5 | 1 | 16 |  | Yes | Known | NA | No | No | No | No | Yes | Yes |  |  | Yes | Yes |  |  | Yes | Yes | left | Thick stalk | 150ml | No |  |  |
| 206 | 2022 | France | 29 | 3 | 2 | 16 |  | NA | Unknown | No | No | No | No | No | No | Yes |  |  |  | Yes |  |  | No | NA | NA | Na | NA | NA |  |  |
| 207 | 2010 | UK | 33 | 3 | 0 | 16 |  | Yes | Unknown | No | Yes | No | Yes | No | No | NA |  |  |  | Yes |  |  | Yes | NA | left | Thick stalk | 3000 | Yes |  | diagnostic laparoscopy at 7th weeks of pregnancy with wrong diagnosis of bicornis, excision of rudimentary honr 3 months later |
| 208 | 2005 | India | 21 | 2 | 1 | 16 |  | No | Unknown | No | Yes | No | No | Yes | No | NA |  |  | No | Yes |  |  | No | No | right | Thick stalk | NA | No |  |  |
| 209 | 2017 | Turquie | 21 | 1 | 0 | 12 |  | No | Unknown | No | No | NO | No | No | Yes | Yes |  |  | yes |  |  |  | No | NA | right | Thin stalk | minimal | No |  | Attempt to terminate the prengnacy failed with misoprostol and curettage |
| 210 | 2013 | India | 22 | 2 | 1 | 17 |  | Yes | Unknown | NA | Yes | No | No | No | No | NA |  |  |  | Yes |  |  | Yes | NA | right | NA | 650 | Yes |  |  |
| 211 | 2010 | Portugal | 26 | 2 | 1 | 17 |  | No | Unknown | No | Yes | No | No | No | NO | NA |  |  |  | Yes |  |  | No | No | right | Thick stalk | NA | No |  |  |
| 212 | 1950 | USA | NA | 2 | 1 | 20 |  | NA | Unknown | no | Yes | No | Yes | No | NA | NA |  |  | Yes |  |  |  | Yes | NA | NA | NA | 2200 | Yes |  |  |
| 213 | 2013 | South Africa | 22 | 1 | 0 | 15 |  | yes | Unknown | no | yes | no | Yes | yes | NA | NA |  |  | yes |  |  |  | Yes | NA | right | Thick stalk | 3000 | yes |  |  |
| 214 | 2002 | Turkey | 22 | 2 | 1 | 15 |  | NA | Known | No | Yes | No | Yes | No | NA | NA |  |  | Yes |  |  |  | Yes | No | right | NA | 3500 | Yes |  |  |
| 215 | 1955 | USA | 35 | 2 | 1 | 18 |  | NA | Unknown | No | Yes | No | Yes | Yes | NA | NA |  |  | Yes |  |  |  | Yes | No | left | Thick stalk | 2000 | Yes |  |  |
| 216 | 2008 | Oman | 24 | 2 | 1 | 14 |  | No | Unknown | No | Yes | No | Yes | Yes | No | NA |  |  | Yes |  |  |  | Yes | NA | NA | NA | 2000 | Yes |  |  |
| 217 | 2011 | India | 22 | 1 | 0 | 14 |  | No | Unknown | No | Yes | NA | Yes | No | No | NA |  |  | Yes |  |  |  | Yes | NA | right | NA | 3500 | Yes |  |  |
| 218 | 2005 | Australia | 29 | 2 | 1 | 16 |  | No | Bicornis | No | Yes | No | Yes | No | No | NA |  |  | Yes |  |  |  | Yes | NA | right | NA | 2000 | Yes |  | Literature review |
| 219 | 2015 | India | 22 | 1 | 0 | 14 |  | No | Unknown | no | Yes | no | no | no | no | na |  |  | yes |  |  |  | Yes | na | left | Thick stalk | 1500 | Yes |  | Heterotopic pregnancy with good outcome |
| 220 | 2018 | Italy | 31 | 2 | 0 | 18 |  | Yes | Unknown | NA | Yes | NA | Yes | No | No | NA |  |  | Yes | Yes |  |  | Yes | NA | left | NA | NA | Yes |  |  |
| 221 | 2004 | India | 22 | 1 | 0 | 20 |  | Yes | Unknown | No | Yes | Yes | Yes | No | No | NA |  |  | Yes |  |  |  | Yes | NA | left | Thick stalk | 1500 | Yes |  |  |
| 222 | 2019 | Pakistan | 20 | 1 | 0 | 17 |  | Yes | Unknown | No | Yes | Yes | Yes | No | No | NA |  |  | Yes |  |  |  | Yes | NA | left | Thick stalk | 1000 | NA |  | explorative Laparotomie |
| 223 | 2018 | Pakistan | 18 | 1 | 0 | 17 |  | no | Unknown | no | Yes | n | Yes | No | No | NA |  |  | Yes |  |  |  | yes | na | right | Thick stalk | 1500 | Yes |  |  |
| 224 | 2011 | Malaysia | 28 | 2 | 1 | 19 |  | No | Unknown | No | Yes | NA | Yes | No | No | NA |  |  | Yes |  |  |  | Yes | NA | right | Thick stalk | 3500 | Yes |  |  |
| 225 | 2000 | Turkey | 21 | 1 | 0 | 13 |  | Yes | Unknown | No | Yes | No | No | No | No | NA |  |  | Yes |  |  |  | Yes | NA | right | Thick stalk | 1500 | Yes |  | Fetus malformation by flap of amniotic membrane |
| 226 | 1992 | Turkey | 20 | 2 | 1 | 16 |  | No | Unknown | No | Yes | Yes | Yes | No | No | NA |  |  | Yes |  |  |  | Yes | NA | right | Thick stalk | NA | Yes |  |  |
| 227 | 2012 | Oman |  | 1 | 0 | 22 |  | No | Unknown | No | yes | no | yes | no | No | NA |  |  | yes |  |  |  | yes | No | left | Thin stalk | 3000 | yes |  |  |
| 228 | 1993 | USA | 20 | 1 | 0 | 23 |  | Yes | Unknown | No | Yes | No | No | No | No | NA |  |  | Yes |  |  |  | Yes | Yes | NA | NA | NA | NA |  | Living infant 444g |
| 229 | 2007 | Pakistan | 36 | 4 | 0 | 13 |  | Yes | Unknown | No | Yes | Yes | No | No | No | NA |  |  | Yes |  |  |  | Yes | Yes | right | Thick stalk | NA | No |  | Laparotomy for appendicitis, bowel covering rudimentary horn rupture |
| 230 | 2001 | Turkey | 24 | 2 | 1 | 14 |  | No | Unknown | No | Yes | NA | Yes | No | No | NA |  |  | Yes |  |  |  | Yes | Yes | right | Thick stalk | 400 | Yes |  |  |
| 231 | 1997 | Turkey | 27 | 3 | 2 | 16 |  | Yes | Unknown | No | Yes | No | Yes | No | No | NA |  |  | Yes |  |  |  | Yes | Yes | right | Thin stalk | 1000 | Yes |  |  |
| 232 | 2021 | Inde | 24 | 1 | 0 | 11 |  | Yes | Unknown | No | Yes | Yes | Yes | No | Yes | NA |  |  | Yes |  |  |  | Yes | NA | right | NA | 1500ml | Yes |  |  |
| 233 | 2017 | Iran | 28 | 2 | 1 | 14 |  | NA | Unknown | No | Yes | NA | Yes | No | Yes | NA |  |  | Yes |  |  |  | Yes | NA | right | Thick stalk | 3000 | Yes |  |  |
| 234 | 2022 | France | 26 | 1 | 0 | 18 |  | NA | Unknown | Yes, renal agenesis | No | No | No | No | No | No |  |  | Yes | Yes |  |  | No | NA | NA | NA | NA | NA |  |  |
| 235 | 2008 | USA | 27 | 3 | 1 | 19 |  | Yes | Unknown | NA | No | NO | NO | No | No | NA |  |  |  | yes |  |  | No | Yes | left | Thick stalk | 200 | No |  | Diagnosis with CT scan, only laaproscopy with operning the rdimentary Horn to developp the fetus |
| 236 | 2010 | India | 21 | 2 | 0 | 28 |  | Yes | Unknown | no left Kidney | Yes | No | Yes | No | No | No | NA |  | Yes |  |  | 28 | Yes | Yes | right | Large stagger | 1500 | Yes |  | Living enfant |
| 237 | 1977 | Canada | 27 | 2 | 1 | 44 |  | Yes | Unknown | No left Kidney | No | Ni | No | No | No | NA | No |  | Yes |  |  | 44 | No | NA | NA | NA | NA | NA |  | Preeclampsy |
| 238 | 1999 | Autralia | 30 | 1 | 0 | 29 |  | Yes | Unknown | No right kidney | Yes | Yes | No | No | No | NA | No |  | Yes |  |  | 29 | Yes | Yes | right | NA | 500 | Yes |  | Rudimentary Horn closed, not removed, living child |
| 239 | 2013 | Portugal | 22 | 1 | 0 | 34 |  | Yes | Unknown | No right Kidney | No | No | No | No | No | NA | Yes |  | Yes |  |  | 34 | No | No | right | Large stagger | minimal | No |  |  |
| 240 | 2015 | India | NA | 3 | 2 | 20 |  | NA | Unknown | NA | Yes | NA | No | No | No | Yes |  |  |  | Yes |  |  | No | NA | NA | NA | NA | NA |  | Torsion of the Horn |
| 241 | 2014 | Oman | 24 | 2 | 1 | 22 |  | No | Unknown | No right kidney | no | no | no | no | yes | yes |  |  | yes |  |  |  | no | NA | right | Thick stalk | minimal | no |  |  |
| 242 | 2013 | India | 20 | 2 | 1 | 20 |  | Yes | Unknown | NA | Yes | No | No | No | No | NA |  |  |  | Yes |  |  | Yes | NA | right | NA | 100 | Yes |  | Low Hb but not by hemoperitoneum, covered rupture by omentum |
| 243 | 2010 | Portugal | 25 | 2 | 1 | 21 |  | No | Didelphys | Left renal agenesis | No | No | No | No | No | Yes |  |  |  | Yes |  |  | No | NA | left | Thick stalk | NA | No |  |  |
| 244 | 2020 | USA | 33 | 3 | 0 | 12 |  | NA | Unknown | none | No | No | No | No | No | No |  |  | Yes |  |  |  | no | Yes | right | NA | minimal | No |  | explorative Laparotomie |
| 245 | 2003 | USA | 25 | 2 | 1 | 19 |  | Yes | Bicornis | right pelvic kidney | Yes | Yes | Yes | No | No | No | No |  | Yes |  |  | 24 | Yes | NA | right | NA | 3000 | NA |  | Resuscitation failure for the fetus |
| 246 | 1993 | Hawai | 26 | 4 | 3 | 24 |  | No | Unknown | small left kidney | No | No | No | No | No | Na | No |  | Yes |  |  | 24 | No | No | left | Thin stagger | NA | No |  |  |
| 247 | 1989 | Lebanon | 23 | 3 | 2 | 28 |  | Yes | No | Spina bifida oculta and double left collecting system | No | No | No | No | No | NA | No |  | Yes |  |  | 28 | No | Yes | right | Large stagger | minimal | No |  | IUGR |
| 248 | 2021 | USA | 26 | 1 | 0 | 23 |  | Yes | Unknown | Vaginal | Yes | No | No | No | No | Yes | Yes |  | Yes, midline |  |  | 25 | No | Yes | right | Thin stagger | NA | No |  | Living enfant |
| 249 | 2002 | Greece | 30 | 2 | 0 | 7 | Yes | Hypoplastic right Horn | No | Yes | Yes | No | Yes | No | No | NA |  |  | Yes |  |  |  | Yes | NA | right | Thick stalk | a lot | Yes |  | Suspected diagnosis at 7. week, Rupture at 20. weeks |
| 250 | 2024 | USA | 39 | 1 | 0 | NA | 5,644 | NA | Unknown | No | No | No | no | No | No | NA |  |  |  | Yes |  |  | NO | NA | left | NA | minimal | No |  | Serie 11 casses |

References :

| 1 | Strohmer, H., Boldizsar, A., & Feichtinger, W. (1992). 'Chemical curettage' using intrauterine methotrexate injection. *Human reproduction (Oxford, England)*, *7*(7), 1027–1028. https://doi.org/10.1093/oxfordjournals.humrep.a137763 |
| --- | --- |
| 2 | Hamai, Y., Fujii, T., Iwasaki, M., Muronosono, E., & Taketani, Y. (1997). A case of pregnancy in a woman with cloacal dysgenesis and a rudimentary uterine horn. *Human reproduction (Oxford, England)*, *12*(5), 1103–1105. https://doi.org/10.1093/humrep/12.5.1103 |
| 3 | Liang, S. T., Woo, J. S., Tang, L. C., & Wong, R. L. (1985). Advanced pregnancy in the rudimentary horn of a bicornuate uterus. *Acta obstetricia et gynecologica Scandinavica*, *64*(5), 447–449. https://doi.org/10.3109/00016348509155165 |
| 4 | Edelman, A. B., Jensen, J. T., Lee, D. M., & Nichols, M. D. (2003). Successful medical abortion of a pregnancy within a noncommunicating rudimentary uterine horn. *American journal of obstetrics and gynecology*, *189*(3), 886–887. https://doi.org/10.1067/s0002-9378(03)00121-2 |
| 5 | Yan C. M. (2010). Laparoscopic management of three rare types of ectopic pregnancy. *Hong Kong medical journal = Xianggang yi xue za zhi*, *16*(2), 132–136. |
| 6 | Perez-Medina, T., García-Andrade, C., & Bajo-Arenas, J. (2009). Advanced pregnancy loss in the rudimentary horn of an undiagnosed unicornuate uterus. *The journal of obstetrics and gynaecology research*, *35*(3), 572–573. https://doi.org/10.1111/j.1447-0756.2008.00978.x |
| 7 | Shamase, N. B., Ntsele, S. J., & Hammond, A. S. (2023). Non-communicating rudimentary horn pregnancy presenting as sudden unexpected maternal death: an autopsy diagnosis. *Forensic science, medicine, and pathology*, *19*(3), 382–387. https://doi.org/10.1007/s12024-022-00504-2 |
| 8 | Chang, J. C., & Lin, Y. C. (1992). Rupture of rudimentary horn pregnancy. *Acta obstetricia et gynecologica Scandinavica*, *71*(3), 235–238. https://doi.org/10.3109/00016349209009926 |
| 9 | Siwatch, S., Mehra, R., Pandher, D. K., & Huria, A. (2013). Rudimentary horn pregnancy: a 10-year experience and review of literature. *Archives of gynecology and obstetrics*, *287*(4), 687–695. https://doi.org/10.1007/s00404-012-2625-7 |
| 10 | Sarikaya, S., & Aybay, M. N. (2022). Uterine rupture of a patient with rudimentary horn pregnancy at 26th gestational weeks. *International journal of surgery case reports*, *94*, 107003. https://doi.org/10.1016/j.ijscr.2022.107003 |
| 11 | Emembolu J. O. (1989). Rudimentary horn pregnancy associated with pre-eclampsia. *International journal of gynaecology and obstetrics: the official organ of the International Federation of Gynaecology and Obstetrics*, *30*(4), 367–370. https://doi.org/10.1016/0020-7292(89)90825-4 |
| 12 | Chopra, S., Suri, V., & Aggarwal, N. (2007). Rudimentary horn pregnancy: prerupture diagnosis and management. *Indian journal of medical sciences*, *61*(1), 28–29. |
| 13 | Shinohara, A., Yamada, A., & Imai, A. (2005). Rupture of noncommunicating rudimentary uterine horn at 27 weeks' gestation with neonatal and maternal survival. *International journal of gynaecology and obstetrics: the official organ of the International Federation of Gynaecology and Obstetrics*, *88*(3), 316–317. https://doi.org/10.1016/j.ijgo.2004.12.023 |
| 14 | Rana, A., Gurung, G., Rawal, S., Bista, K. D., Adhukari, S., & Ghimire, R. K. (2008). Surviving 27 weeks fetus expelled out of the ruptured rudimentary horn and detected a month later as a secondary abdominal pregnancy. *The journal of obstetrics and gynaecology research*, *34*(2), 247–251. https://doi.org/10.1111/j.1447-0756.2008.00763.x |
| 15 | Nahum G. G. (1997). Rudimentary uterine horn pregnancy. A case report on surviving twins delivered eight days apart. *The Journal of reproductive medicine*, *42*(8), 525–532. |
| 16 | Bidiga, S., Henry, K., Augustino, O., Mujuni, F., Matovelo, D., Ndaboine, E., Kihunrwa, A., & Kiritta, R. (2023). Rudimentary horn pregnancy, a differential diagnosis of an intraabdominal pregnancy: a case report. *Journal of medical case reports*, *17*(1), 210. https://doi.org/10.1186/s13256-023-03882-5 |
| 17 | Zaidi, J., & Carr, J. (1994). Rupture of pregnant rudimentary uterine horn with fetal salvage. *Acta obstetricia et gynecologica Scandinavica*, *73*(4), 359–360. https://doi.org/10.3109/00016349409015781 |
| 18 | Juneja, S. K., Gupta, S., Tandon, P., & Gumber, N. (2017). Rupture of Noncommunicating Rudimentary Horn of Uterus. *International journal of applied & basic medical research*, *7*(2), 146–147. https://doi.org/10.4103/ijabmr.IJABMR_112_16 |
| 19 | Fekih, M., Memmi, A., Nouri, S., Ben Regaya, L., Bouguizene, S., Essaidi, H., Chaieb, A., Bibi, M., Sboui, H., & Khairi, H. (2009). Asymptomatic horn rudimentary pregnant uterine rupture with a viable fetus. *La Tunisie medicale*, *87*(9), 633–636. |
| 20 | Dadgar, S., Mahmoudinia, M., & Davaryari, N. (2023). Pregnancy in the Unicornuate Uterus and Non-Communicating Rudimentary Horn. *Iranian journal of medical sciences*, *48*(6), 612–614. https://doi.org/10.30476/IJMS.2023.97859.2971 |
| 21 | Al Qarni, A. A., Al-Braikan, N., Al-Hanbali, M. M., & Alharmaly, A. H. (2017). Rupture rudimentary horn pregnancy at 31 week. *Saudi medical journal*, *38*(2), 201–203. https://doi.org/10.15537/smj.2017.2.16016 |
| 22 | Sinha, R., Sachan, S., & Khanna, A. (2011). Successful outcome in preeclamptic rudimentary horn pregnancy. *Journal of the Turkish German Gynecological Association*, *12*(1), 53–55. https://doi.org/10.5152/jtgga.2011.12 |
| 23 | Mengistu, K., Bobe, T., Tilahun, G., Kifle, K., & Geleta, D. (2020). Rudimentary Horn Pregnancy Diagnosed after Laparotomy. *Case reports in obstetrics and gynecology*, *2020*, 5816487. https://doi.org/10.1155/2020/5816487 |
| 24 | Cuppett, C. D., Stitely, M. L., & Toffle, R. C. (2011). Unruptured 32-week rudimentary horn pregnancy presenting as right upper quadrant pain. *The West Virginia medical journal*, *107*(4), 8–10. |
| 25 | Solomon, T., Jemal, E., Ahmed, K., Tesfaye, M., & Alemayehu, A. (2024). Extremely uncommon torsion and communicating ruptured rudimentary horn pregnancy at third trimester: A case report. *SAGE open medical case reports*, *12*, 2050313X241272629. https://doi.org/10.1177/2050313X241272629 |
| 26 | Shin, J. W., & Kim, H. J. (2005). Case of live birth in a non-communicating rudimentary horn pregnancy. *The journal of obstetrics and gynaecology research*, *31*(4), 329–331. https://doi.org/10.1111/j.1447-0756.2005.00296.x |
| 27 | Xu, S., Zhang, J., Yue, S., Qian, J., Yang, L., Xu, Y. Z., & Zhang, J. (2024). A case of rudimentary uterine horn pregnancy complicated with placental implantation in later stages of pregnancy. *Quantitative imaging in medicine and surgery*, *14*(1), 1167–1172. https://doi.org/10.21037/qims-23-889 |
| 28 | Nanda, S., Dahiya, K., Sharma, N., Aggarwal, D., Sighal, S. R., & Sangwan, N. (2009). Successful twin pregnancy in a unicornuate uterus with one fetus in the non-communicating rudimentary horn. *Archives of gynecology and obstetrics*, *280*(6), 993–995. https://doi.org/10.1007/s00404-009-1028-x |
| 29 | Elito Júnior, J., Goldman, S. M., Castro, P. T., Werner, H., Sanchez, V. H. S., & Araujo Júnior, E. (2024). Heterotopic twin pregnancy in unicornuate uterus and non-communicating rudimentary horn with survival of both fetuses: Magnetic resonance imaging and 3D reconstructions findings. *Journal of clinical ultrasound : JCU*, *52*(8), 1193–1197. https://doi.org/10.1002/jcu.23747 |
| 30 | Taifour, W., Aljammal, G., Bhsass, R., Almnashef, R., Alshikh, Y., & Adwan, D. (2024). Rupture of non-communicating rudimentary horn at 35 weeks ending with a live birth: A case report. *International journal of surgery case reports*, *119*, 109641. https://doi.org/10.1016/j.ijscr.2024.109641 |
| 31 | Akhtar A. Z. (1988). Term pregnancy in a rudimentary horn of a bicornuate uterus with foetal salvage: a case report. *Asia-Oceania journal of obstetrics and gynaecology*, *14*(2), 143–146. https://doi.org/10.1111/j.1447-0756.1988.tb00085.x |
| 32 | Chou, M. M., Ho, E. S., Lin, S. K., Yang, S. J., Lee, Y. H., Huang, P. C., & Chang, S. M. (1999). Term pregnancy in a noncommunicating rudimentary horn of an unicornuate uterus: a case report. *Zhonghua yi xue za zhi = Chinese medical journal; Free China ed*, *62*(6), 383–387. |
| 33 | Iyoke, C., Okafor, C., Ugwu, G., & Oforbuike, C. (2014). Live Birth Following a Term Pregnancy in a Non-communicating Rudimentary Horn of a Unicornuate Uterus. *Annals of medical and health sciences research*, *4*(1), 126–128. https://doi.org/10.4103/2141-9248.126622 |
| 34 | Cheng, C., Tang, W., Zhang, L., Luo, M., Huang, M., Wu, X., & Wan, G. (2015). Unruptured pregnancy in a noncommunicating rudimentary horn at 37 weeks with a live fetus: a case report. *Journal of biomedical research*, *29*(1), 83–86. https://doi.org/10.7555/JBR.29.20130089 |
| 35 | Patra, S., Puri, M., Trivedi, S. S., Yadav, R., & Bali, J. (2007). Unruptured term pregnancy with a live fetus with placenta percreta in a non-communicating rudimentary horn. *Congenital anomalies*, *47*(4), 156–157. https://doi.org/10.1111/j.1741-4520.2007.00163.x |
| 36 | Shrivastava, N., Yadav, S., & Shrivastava, V. (2015). Term Pregnancy with a Live Fetus in Non-communicating Rudimentary Horn with Placenta Percreta. *Journal of obstetrics and gynaecology of India*, *65*(5), 339–341. https://doi.org/10.1007/s13224-014-0653-x |
| 37 | Acharya, S., & Barnick, C. (2003). Retained pregnancy for five years in a rudimentary uterine horn. *Acta obstetricia et gynecologica Scandinavica*, *82*(4), 387–388. https://doi.org/10.1034/j.1600-0412.2003.00100.x |
| 38 | Pal, K., Majumdar, S., & Mukhopadhyay, S. (2006). Rupture of rudimentary uterine horn pregnancy at 37 weeks gestation with fetal survival. *Archives of gynecology and obstetrics*, *274*(5), 325–326. https://doi.org/10.1007/s00404-006-0170-y |
| 39 | Rutten, C., Khadam, L., Picamoles, P., Fokou-Soh, R. M., Alperin, E., & Belaidi, N. (2017). Lithopedion developed in a non-communicating rudimentary uterine horn: CT features. *Diagnostic and interventional imaging*, *98*(11), 817–818. https://doi.org/10.1016/j.diii.2017.04.007 |
| 40 | Upadhyaya I. (2011). Non-communicating rudimentary uterine horn pregnancy. *JNMA; journal of the Nepal Medical Association*, *51*(184), 199–202. |
| 41 | O'Grady, J. P., & Salem, F. A. (1978). Rudimentary horn pregnancy with neonatal and maternal survival. *Journal of the National Medical Association*, *70*(11), 863–866. |
| 42 | Amritha, B., Sumangali, T., Priya, B., Deepak, S., & Sharadha, R. (2009). A rare case of term viable secondary abdominal pregnancy following rupture of a rudimentary horn: a case report. *Journal of medical case reports*, *3*, 38. https://doi.org/10.1186/1752-1947-3-38 |
| 43 | Aminu, M. B., Sania, I., & Khairunnaesa, M. (2023). Post-dated Breech Pregnancy in a Non-obviously Communicating Rudimentary Horn of a Bicornuate Uterus Requiring Hemi-hysterectomy. *Journal of the West African College of Surgeons*, *13*(1), 111–113. https://doi.org/10.4103/jwas.jwas_194_22 |
| 44 | Goel, P., Saha, P. K., Mehra, R., & Huria, A. (2007). Unruptured postdated pregnancy with a live fetus in a noncommunicating rudimentary horn. *Indian journal of medical sciences*, *61*(1), 23–27. |
| 45 | Souza, C. S., Dorneles, G. G., Mendonça, G. N., Santos, C. M. D., Gallarreta, F. M. P., & Konopka, C. K. (2017). Pregnancy in Non-Communicating Unicornuate Uterus: Diagnosis Difficulty and Outcomes - a Case Report. Gestação em útero unicorno não comunicante: dificuldade diagnóstica e desfechos – relato de caso. *Revista brasileira de ginecologia e obstetricia : revista da Federacao Brasileira das Sociedades de Ginecologia e Obstetricia*, *39*(11), 640–644. https://doi.org/10.1055/s-0037-1607046 |
| 46 | Grover, V., Dhall, K., Dhall, G. I., & Dogra, M. (1986). Problems of rudimentary horn pregnancy. *Gynecologic and obstetric investigation*, *22*(1), 52–55. https://doi.org/10.1159/000298890 |
| 47 | Grover, V., Dhall, K., Dhall, G. I., & Dogra, M. (1986). Problems of rudimentary horn pregnancy. *Gynecologic and obstetric investigation*, *22*(1), 52–55. https://doi.org/10.1159/000298890 |
| 48 | Grover, V., Dhall, K., Dhall, G. I., & Dogra, M. (1986). Problems of rudimentary horn pregnancy. *Gynecologic and obstetric investigation*, *22*(1), 52–55. https://doi.org/10.1159/000298890 |
| 49 | Ngieng L. N. (1970). A case of rudimentary horn pregnancy that reached term. *The Medical journal of Malaya*, *25*(1), 65–67. |
| 50 | White C. (1919). A Case of Full-time Pregnancy in a Rudimentary Uterine Horn. *Proceedings of the Royal Society of Medicine*, *12*(Obstet Gynaecol Sect), 138–140. https://doi.org/10.1177/003591571901200929 |
| 51 | Jihong, L., Siow, A., & Chern, B. (2009). Laparoscopic excision of rudimentary horn pregnancy in a patient with previous caesarean section. *Archives of gynecology and obstetrics*, *279*(3), 403–405. https://doi.org/10.1007/s00404-008-0726-0 |
| 52 | Chang, W. F., Lin, H. H., Ho, H. N., Sheu, B. C., Huang, S. C., & Lee, T. Y. (1994). Ultrasound diagnosis of rudimentary uterine horn pregnancy in fourteen weeks of gestation: a case report. *Asia-Oceania journal of obstetrics and gynaecology*, *20*(3), 279–282. https://doi.org/10.1111/j.1447-0756.1994.tb00470.x |
| 53 | Wahlén T. (1972). Pregnancy in non-communicating rudimentary uterine horn. *Acta obstetricia et gynecologica Scandinavica*, *51*(2), 155–160. https://doi.org/10.3109/00016347209154011 |
| 54 | Wahlén T. (1972). Pregnancy in non-communicating rudimentary uterine horn. *Acta obstetricia et gynecologica Scandinavica*, *51*(2), 155–160. https://doi.org/10.3109/00016347209154011 |
| 55 | De NICOLA, R. R., & PETERSEN, M. R. (1947). Pregnancy in a rudimentary horn of the uterus. *American journal of surgery*, *73*(3), 381–384. https://doi.org/10.1016/0002-9610(47)90349-8 |
| 56 | Banister J. B. (1937). Intra- and Extra-uterine Pregnancy. Excision of Extra-uterine Pregnancy in the Fourth Month. together with the Rudimentary Horn of the Uterus, followed by Full-term Delivery of the Intra-uterine Pregnancy. *Proceedings of the Royal Society of Medicine*, *30*(5), 562. https://doi.org/10.1177/003591573703000513 |
| 57 | NAOR S. L. (1964). PREGNANCY IN A RUDIMENTARY HORN OF THE UTERUS DIAGNOSED BEFORE OPERATION. *American journal of obstetrics and gynecology*, *88*, 131–132. https://doi.org/10.1016/0002-9378(64)90241-8 |
| 58 | GERGELY, E., & MASON, D. J. (1959). Pregnancy in a noncommunicating rudimentary horn. Report of a case. *American journal of obstetrics and gynecology*, *78*, 1202–1204. https://doi.org/10.1016/0002-9378(59)90576-9 |
| 59 | Waters H. (1944). Pregnancy in a Rudimentary Uterine Horn. *The Indian medical gazette*, *79*(8), 355–356. |
| 60 | Wahlén T. (1972). Pregnancy in non-communicating rudimentary uterine horn. *Acta obstetricia et gynecologica Scandinavica*, *51*(2), 155–160. https://doi.org/10.3109/00016347209154011 |
| 61 | Goel, J. K., & Chandrashekhar, M. (2000). PREGNANCY IN A RUDIMENTARY HORN OF BICORNUATE UTERUS. *Medical journal, Armed Forces India*, *56*(4), 351–352. https://doi.org/10.1016/S0377-1237(17)30231-9 |
| 62 | Monacci, F., Lanfredini, N., Zandri, S., Strigini, F., Luchi, C., Giannini, A., & Simoncini, T. (2018). Diagnosis and laparoscopic management of a 5-week ectopic pregnancy in a rudimentary uterine horn: A case report. *Case reports in women's health*, *21*, e00088. https://doi.org/10.1016/j.crwh.2018.e00088 |
| 63 | Tolani, A. D., Kadambari, Deenadayal, A., Donthi, S., Yellenki, I. R., & Deenadayal, M. (2018). Timely Identification of Pregnancy in Noncommunicating Horn of Unicornuate Uterus by Three-Dimensional Transvaginal Ultrasonography. *Journal of clinical imaging science*, *8*, 39. https://doi.org/10.4103/jcis.JCIS_25_18 |
| 64 | Upadhyaya I. (2011). Non-communicating rudimentary uterine horn pregnancy. *JNMA; journal of the Nepal Medical Association*, *51*(184), 199–202. |
| 65 | Moawad, G. N., & Abi Khalil, E. D. (2016). A Case of Recurrent Rudimentary Horn Ectopic Pregnancies Managed by Methotrexate Therapy and Laparoscopic Excision of the Rudimentary Horn. *Case reports in obstetrics and gynecology*, *2016*, 5747524. https://doi.org/10.1155/2016/5747524 |
| 66 | Rodrigues, Â., Neves, A. R., Castro, M. G., Branco, M., Geraldes, F., & Águas, F. (2019). Successful management of a rudimentary uterine horn ectopic pregnancy by combining methotrexate and surgery: A case report. *Case reports in women's health*, *24*, e00158. https://doi.org/10.1016/j.crwh.2019.e00158 |
| 67 | Dhanawat, J., Pape, J., Stuhlmann-Laeisz, C., Maass, N., Freytag, D., Gitas, G., & Alkatout, I. (2021). Ectopic pregnancy in noncommunicating horn of unicornuate uterus: 3D-ultrasound and primary laparoscopic management. *Clinical case reports*, *9*(5), e04261. https://doi.org/10.1002/ccr3.4261 |
| 68 | Upadhyaya I. (2011). Non-communicating rudimentary uterine horn pregnancy. *JNMA; journal of the Nepal Medical Association*, *51*(184), 199–202. |
| 69 | Jiang, Y., Zhong, Y., Dong, L., Zhan, L., Li, P., Guo, F., & Huang, Z. (2022). Diagnosis and management of non-communicating rudimentary horn pregnancy. *Ginekologia polska*, 10.5603/GP.a2021.0168. Advance online publication. https://doi.org/10.5603/GP.a2021.0168 |
| 70 | Rivera Casul, G., & Gallant, T. (2025). Non-Communicating Rudimentary Horn Pregnancy. *Journal of minimally invasive gynecology*, S1553-4650(25)00093-7. Advance online publication. https://doi.org/10.1016/j.jmig.2025.03.008 |
| 71 | Andolf, E., Helm, G., Svalenius, E., & Weström, L. (1988). Seventeen week pregnancy in a rudimentary uterine horn revealed at routine ultrasonography. *Acta obstetricia et gynecologica Scandinavica*, *67*(4), 379–380. |
| 72 | Mohammed, A. A., Abdelfattah, A. I., Ali, M. M., & Elmubarak, L. A. (2023). Intact non-communicating rudimentary horn pregnancy in a patient with a history of two cesarean sections: A case report. *Case reports in women's health*, *41*, e00570. https://doi.org/10.1016/j.crwh.2023.e00570 |
| 73 | Sönmezer, M., Taskin, S., Atabekoğlu, C., Güngör, M., & Unlü, C. (2006). Laparoscopic management of rudimentary uterine horn pregnancy: case report and literature review. *JSLS : Journal of the Society of Laparoendoscopic Surgeons*, *10*(3), 396–399. |
| 74 | Adolph, A. J., & Gilliland, G. B. (2002). Fertility following laparoscopic removal of rudimentary horn with an ectopic pregnancy. *Journal of obstetrics and gynaecology Canada : JOGC = Journal d'obstetrique et gynecologie du Canada : JOGC*, *24*(7), 575–576. https://doi.org/10.1016/s1701-2163(16)31061-1 |
| 75 | Shiber, L. J., & Biscette, S. (2015). Laparoscopic Management of a Rudimentary Horn Pregnancy. *Journal of minimally invasive gynecology*, *22*(6S), S153. https://doi.org/10.1016/j.jmig.2015.08.569 |
| 76 | Numa, F., Ogata, H., Murakami, A., Sase, M., Nakamura, Y., Takasugi, N., & Kato, H. (1995). Adenomyosis in a rudimentary uterine horn with intrauterine fetal death: a case report. *Journal of obstetrics and gynaecology (Tokyo, Japan)*, *21*(3), 289–292. https://doi.org/10.1111/j.1447-0756.1995.tb01012.x |
| 77 | Baughn, M. R., Vaux, K., & Masliah, E. (2010). Placenta accreta in a separate uterine horn. *Pediatric and developmental pathology : the official journal of the Society for Pediatric Pathology and the Paediatric Pathology Society*, *13*(1), 63–65. https://doi.org/10.2350/09-03-0627-CR.1 |
| 78 | Hamet, B., Hoeffel, C., Fague, V., Lucot, J. P., Pagès-Bouic, E., Rousset, P., Graesslin, O., Bazot, M., & Poncelet, E. (2022). Pregnancy in a rudimentary horn: multicenter's MRI features of a rare condition. *Abdominal radiology (New York)*, *47*(12), 4195–4204. https://doi.org/10.1007/s00261-022-03658-3 |
| 79 | Krishnan, M., Parker, V. L., Baxter, A. J., Jha, S., Marrappan, B., & Ola, B. (2024). Early first trimester diagnosis and total laparoscopic management of rudimentary uterine horn pregnancy. *Radiology case reports*, *19*(12), 6487–6490. https://doi.org/10.1016/j.radcr.2024.08.137 |
| 80 | Sevtap, H. K., Aral, A. M., & Sertac, B. (2007). An early diagnosis and successful local medical treatment of a rudimentary uterine horn pregnancy: a case report. *Archives of gynecology and obstetrics*, *275*(4), 297–298. https://doi.org/10.1007/s00404-006-0232-1 |
| 81 | Zhang, D. D., Gao, Y., Lang, J. H., & Zhu, L. (2018). Diagnosis and Treatment of Rudimentary Horn Pregnancy: Analysis of Eleven Cases. *Chinese medical journal*, *131*(24), 3012–3014. https://doi.org/10.4103/0366-6999.247200 |
| 82 | Yahata, T., Kurabayashi, T., Ueda, H., Kodama, S., Chihara, T., & Tanaka, K. (1998). Laparoscopic management of rudimentary horn pregnancy. A case report. *The Journal of reproductive medicine*, *43*(3), 223–226. |
| 83 | Herchelroath, D., Miller, J. L., & Wang, K. C. (2018). Novel Management of Ectopic Pregnancy in a Noncommunicating Rudimentary Horn of a Unicornuate Uterus. *The Journal of the American Osteopathic Association*, *118*(9), 623–626. https://doi.org/10.7556/jaoa.2018.137 |
| 84 | Isono, W., Tsuchiya, A., Honda, M., Saito, A., Tsuchiya, H., Matsuyama, R., Fujimoto, A., & Nishii, O. (2022). Successful Management of a Noncommunicating Rudimentary Uterine Horn Pregnancy by Laparoscopic Surgery: A Case Report and Literature Review. *Gynecology and minimally invasive therapy*, *11*(1), 7–16. https://doi.org/10.4103/GMIT.GMIT_157_20 |
| 85 | Yahata, T., Kurabayashi, T., Ueda, H., Kodama, S., Chihara, T., & Tanaka, K. (1998). Laparoscopic management of rudimentary horn pregnancy. A case report. *The Journal of reproductive medicine*, *43*(3), 223–226. |
| 86 | Taori, K., Saha, B. K., Shah, D., Khadaria, N., Jadhav, V., & Jawale, R. (2008). Sonographic diagnosis of uncomplicated first-trimester pregnancy in the rudimentary horn of a unicornuate uterus. *Journal of clinical ultrasound : JCU*, *36*(1), 45–47. https://doi.org/10.1002/jcu.20337 |
| 87 | Zhang, D. D., Gao, Y., Lang, J. H., & Zhu, L. (2018). Diagnosis and Treatment of Rudimentary Horn Pregnancy: Analysis of Eleven Cases. *Chinese medical journal*, *131*(24), 3012–3014. https://doi.org/10.4103/0366-6999.247200 |
| 88 | Ekpe, E., Garg, N., Lui, P., Sheran, J., & Chaudhari, A. (2023). Laparoscopic Management of an Advanced Nonruptured Ectopic Pregnancy in a Rudimentary Uterine Horn. *Journal of minimally invasive gynecology*, *30*(3), 169–170. https://doi.org/10.1016/j.jmig.2022.12.004 |
| 89 | Ma, Y. C., & Law, K. S. (2022). Pregnancy in a Non-Communicating Rudimentary Horn of Unicornuate Uterus. *Diagnostics (Basel, Switzerland)*, *12*(3), 759. https://doi.org/10.3390/diagnostics12030759 |
| 90 | Henriet, E., Roman, H., Zanati, J., Lebreton, B., Sabourin, J. C., & Loic, M. (2008). Pregnant noncommunicating rudimentary uterine horn with placenta percreta. *JSLS : Journal of the Society of Laparoendoscopic Surgeons*, *12*(1), 101–103. |
| 91 | Wang, Y., Yu, F., & Zeng, L. Q. (2015). Ectopic Pregnancy in Uncommon Implantation Sites: Intramural Pregnancy and Rudimentary Horn Pregnancy. *Case reports in obstetrics and gynecology*, *2015*, 536498. https://doi.org/10.1155/2015/536498 |
| 92 | Hamet, B., Hoeffel, C., Fague, V., Lucot, J. P., Pagès-Bouic, E., Rousset, P., Graesslin, O., Bazot, M., & Poncelet, E. (2022). Pregnancy in a rudimentary horn: multicenter's MRI features of a rare condition. *Abdominal radiology (New York)*, *47*(12), 4195–4204. https://doi.org/10.1007/s00261-022-03658-3 |
| 93 | Ji, J., Tan, L., & Lv, K. (2024). Imaging diagnosis of rudimentary horn pregnancy: a case report. *AME case reports*, *8*, 34. https://doi.org/10.21037/acr-23-164 |
| 94 | Anwari L. (2021). Prerupture diagnosis of a pregnant rudimentary uterine horn. *Radiology case reports*, *16*(4), 764–768. https://doi.org/10.1016/j.radcr.2021.01.029 |
| 95 | Si, M., Li, P., Yuan, Z., Ma, H., Cui, B., & Kong, B. (2017). An unexpected invasive hydatidiform mole in a rudimentary uterine horn: A case report. *Oncology letters*, *14*(3), 2808–2812. https://doi.org/10.3892/ol.2017.6493 |
| 96 | Singh, R., Himabindu, N., Jayavani, R. L., & Gajalakshmi, R. (2016). Unruptured Pregnancy in Rudimentary Horn Presenting as Hemoperitoneum. *Journal of obstetrics and gynaecology of India*, *66*(Suppl 2), 626–628. https://doi.org/10.1007/s13224-016-0891-1 |
| 97 | Smolders, D., Deckers, F., Pouillon, M., Vanderheyden, T., Vanderheyden, J., & De Schepper, A. (2002). Ectopic pregnancy within a rudimentary horn in a case of unicornuate uterus. *European radiology*, *12*(1), 121–124. https://doi.org/10.1007/s003300100939 |
| 98 | Cobec, I. M., Seropian, P., & Rempen, A. (2019). Pregnancy in a non-communicating rudimentary horn of a unicornuate uterus. *Hippokratia*, *23*(2), 92–94. |
| 99 | Ercan, C. M., Coksuer, H., Pehlivan, H., Ceyhan, S. T., Alanbay, I., & Baser, I. (2012). Saline infusion sonography in the accurate diagnosis of a rudimentary horn pregnancy. *Journal of obstetrics and gynaecology : the journal of the Institute of Obstetrics and Gynaecology*, *32*(3), 311–312. https://doi.org/10.3109/01443615.2011.647735 |
| 100 | Lawhon, B. P., Wax, J. R., & Dufort, R. T. (1998). Rudimentary uterine horn pregnancy diagnosed with magnetic resonance imaging. *Obstetrics and gynecology*, *91*(5 Pt 2), 869. https://doi.org/10.1016/s0029-7844(97)00471-7 |
| 101 | Zhang, D. D., Gao, Y., Lang, J. H., & Zhu, L. (2018). Diagnosis and Treatment of Rudimentary Horn Pregnancy: Analysis of Eleven Cases. *Chinese medical journal*, *131*(24), 3012–3014. https://doi.org/10.4103/0366-6999.247200 |
| 102 | Tsafrir, A., Rojansky, N., Sela, H. Y., Gomori, J. M., & Nadjari, M. (2005). Rudimentary horn pregnancy: first-trimester prerupture sonographic diagnosis and confirmation by magnetic resonance imaging. *Journal of ultrasound in medicine : official journal of the American Institute of Ultrasound in Medicine*, *24*(2), 219–223. https://doi.org/10.7863/jum.2005.24.2.219 |
| 103 | Zhang, D. D., Gao, Y., Lang, J. H., & Zhu, L. (2018). Diagnosis and Treatment of Rudimentary Horn Pregnancy: Analysis of Eleven Cases. *Chinese medical journal*, *131*(24), 3012–3014. https://doi.org/10.4103/0366-6999.247200 |
| 104 | Terzi, H., Yavuz, A., Demirtaş, Ö., & Kale, A. (2014). Rudimentary horn pregnancy in the first trimester; importance of ultrasound and clinical suspicion in early diagnosis: A case report. *Turkish journal of obstetrics and gynecology*, *11*(3), 189–192. https://doi.org/10.4274/tjod.10437 |
| 105 | Dicker, D., Nitke, S., Shoenfeld, A., Fish, B., Meizner, I., & Ben-Rafael, Z. (1998). Laparoscopic management of rudimentary horn pregnancy. *Human reproduction (Oxford, England)*, *13*(9), 2643–2644. https://doi.org/10.1093/humrep/13.9.2643 |
| 106 | Tsafrir, A., Rojansky, N., Sela, H. Y., Gomori, J. M., & Nadjari, M. (2005). Rudimentary horn pregnancy: first-trimester prerupture sonographic diagnosis and confirmation by magnetic resonance imaging. *Journal of ultrasound in medicine : official journal of the American Institute of Ultrasound in Medicine*, *24*(2), 219–223. https://doi.org/10.7863/jum.2005.24.2.219 |
| 107 | van Esch, E. M., Lashley, E. E., Berning, B., & de Kroon, C. D. (2010). The value of hysteroscopy in the diagnostic approach to a rudimentary horn pregnancy. *BMJ case reports*, *2010*, bcr0820103229. https://doi.org/10.1136/bcr.08.2010.3229 |
| 108 | Siragusa, M. J., Aspiazu, A. S. N., Marquez, A. A., Chacon, C. R., Otaño, L., & Saadi, J. M. (2024). Laparoscopic Approach for Ectopic Pregnancy in a Rudimentary Horn: Stepwise Demonstration of Surgical Technique. *Journal of minimally invasive gynecology*, *31*(7), 556. https://doi.org/10.1016/j.jmig.2024.03.008 |
| 109 | Kriplani, A., Relan, S., Mittal, S., & Buckshee, K. (1995). Pre-rupture diagnosis and management of rudimentary horn pregnancy in the first trimester. *European journal of obstetrics, gynecology, and reproductive biology*, *58*(2), 203–205. https://doi.org/10.1016/0028-2243(94)01956-8 |
| 110 | Essebbagh, Y., Errmili, K., Belouazza, F. Z., Slaoui, A., Zeraidi, N., & Baidada, A. (2025). Early first trimester diagnosis and management of rudimentary horn pregnancy: A rare case report. *Radiology case reports*, *20*(9), 4722–4726. https://doi.org/10.1016/j.radcr.2025.05.074 |
| 111 | Park, J. K., & Dominguez, C. E. (2007). Combined medical and surgical management of rudimentary uterine horn pregnancy. *JSLS : Journal of the Society of Laparoendoscopic Surgeons*, *11*(1), 119–122. |
| 112 | Nelson, K. L., Osborne, C., & McQuillan, S. K. (2022). Pregnancy and Placenta Increta in a Noncommunicating Uterine Horn. *Journal of pediatric and adolescent gynecology*, *35*(1), 101–103. https://doi.org/10.1016/j.jpag.2021.08.003 |
| 113 | Kumar, N., Das, V., Pandey, A., & Agrawal, S. (2018). Torsion and rupture of a non-communicating rudimentary horn in a 17-week gestation in a 16-year-old girl: lessons learnt. *BMJ case reports*, *2018*, bcr2017222073. https://doi.org/10.1136/bcr-2017-222073 |
| 114 | JOHN A. H. (1961). Uterus bicornis unicollis complicated by pregnancy in a rudimentary horn and with subsequent normal pregnancy. *The Journal of obstetrics and gynaecology of the British Empire*, *68*, 297–298. https://doi.org/10.1111/j.1471-0528.1961.tb02728.x |
| 115 | Scanzoni (1854). Case of Pregnancy in a Rudimentary Horn of the Uterus, with Probable Advance of the Ovum from the Right Ovary into the Left Horn of the Uterus. *Edinburgh medical and surgical journal*, *81*(198), 201–202. |
| 116 | Sahakian V. (1992). Rupture of a rudimentary horn pregnancy with a combined intrauterine pregnancy. A case report. *The Journal of reproductive medicine*, *37*(3), 283–284. |
| 117 | Willett A. (1909). Pregnancy in a Rudimentary Horn of a Bicornate Uterus. *Proceedings of the Royal Society of Medicine*, *2*(Obstet Gynaecol Sect), 342–344. https://doi.org/10.1177/003591570900200858 |
| 118 | SALEH J. S. (1955). Rupture of a four month gravid rudimentary horn of a bicornate uterus. *American journal of obstetrics and gynecology*, *70*(2), 426–427. https://doi.org/10.1016/s0002-9378(16)37690-6 |
| 119 | Harzallah, H., Ben Khelil, M., & Hamdoun, M. (2018). A Case of Death Resulting from the Complications of a Rudimentary Uterine Horn Pregnancy. *Journal of obstetrics and gynaecology Canada : JOGC = Journal d'obstetrique et gynecologie du Canada : JOGC*, *40*(2), 139. https://doi.org/10.1016/j.jogc.2017.11.029 |
| 120 | Pongsuthirak, P., Tongsong, T., & Srisomboon, J. (1993). Rupture of a noncommunicating rudimentary uterine horn pregnancy with a combined intrauterine pregnancy. *International journal of gynaecology and obstetrics: the official organ of the International Federation of Gynaecology and Obstetrics*, *41*(2), 185–187. https://doi.org/10.1016/0020-7292(93)90706-3 |
| 121 | GOLDMAN, J. A., & ECKERLING, B. (1959). An unusual case of rupture of a pregnant rudimentary horn of a bicornuate uterus. *American journal of obstetrics and gynecology*, *78*, 1205–1207. https://doi.org/10.1016/0002-9378(59)90577-0 |
| 122 | Jansch R. (1874). A Case of Pregnancy in a Rudimentary Horn of the Uterus; Death by Hæmorrhage; Transmigratio Seminis Extrauterina. *Edinburgh medical journal*, *20*(1), 81. |
| 123 | CARPENTER, R. J., Jr, & JAMESON, W. J. (1952). Uterus bicornis unicollis with rudimentary horn. *American journal of obstetrics and gynecology*, *63*(1), 206–208. https://doi.org/10.1016/s0002-9378(16)39006-8 |
| 124 | Tang, R., Sheng, Y., & Chen, Z. J. (2004). Rupture of pregnancy in a communicating rudimentary uterine horn after in vitro fertilization and embryo transfer. *International journal of gynaecology and obstetrics: the official organ of the International Federation of Gynaecology and Obstetrics*, *86*(3), 394–395. https://doi.org/10.1016/j.ijgo.2004.04.043 |
| 125 | Zhang, D. D., Gao, Y., Lang, J. H., & Zhu, L. (2018). Diagnosis and Treatment of Rudimentary Horn Pregnancy: Analysis of Eleven Cases. *Chinese medical journal*, *131*(24), 3012–3014. https://doi.org/10.4103/0366-6999.247200 |
| 126 | Torbe, E., & Hon, M. S. (2013). Subsequent pregnancy after a ruptured rudimentary uterine horn pregnancy. *European journal of obstetrics, gynecology, and reproductive biology*, *166*(1), 115. https://doi.org/10.1016/j.ejogrb.2012.10.007 |
| 127 | Peters, S. N., Fuller, S. M., & Schmiedecke, S. S. (2025). Laparoscopic endoloop excision of noncommunicating rudimentary uterine horn ectopic pregnancy in the first trimester. *American journal of obstetrics and gynecology*, *232*(3), 330–331. https://doi.org/10.1016/j.ajog.2024.10.026 |
| 128 | Samuels, T. A., & Awonuga, A. (2005). Second-trimester rudimentary uterine horn pregnancy: rupture after labor induction with misoprostol. *Obstetrics and gynecology*, *106*(5 Pt 2), 1160–1162. https://doi.org/10.1097/01.AOG.0000182989.06439.68 |
| 129 | Khanapure, A., Aravind, S., Lawley, R., & Verwood, G. (2005). Rupture of a pregnancy in the rudimentary communicating horn of the uterus. *Journal of obstetrics and gynaecology : the journal of the Institute of Obstetrics and Gynaecology*, *25*(3), 310–311. https://doi.org/10.1080/01443610500105688 |
| 130 | Panayotidis, C., Abdel-Fattah, M., & Leggott, M. (2004). Rupture of rudimentary uterine horn of a unicornuate uterus at 15 weeks' gestation. *Journal of obstetrics and gynaecology : the journal of the Institute of Obstetrics and Gynaecology*, *24*(3), 323–324. https://doi.org/10.1080/01443610410001661057 |
| 131 | Chin, K. A., Wee, L., & Penketh, R. J. (1998). Laparoscopic management of a rudimentary uterine horn pregnancy. *Journal of obstetrics and gynaecology : the journal of the Institute of Obstetrics and Gynaecology*, *18*(5), 498. https://doi.org/10.1080/01443619866949 |
| 132 | Zhang, D. D., Gao, Y., Lang, J. H., & Zhu, L. (2018). Diagnosis and Treatment of Rudimentary Horn Pregnancy: Analysis of Eleven Cases. *Chinese medical journal*, *131*(24), 3012–3014. https://doi.org/10.4103/0366-6999.247200 |
| 133 | Zhang, D. D., Gao, Y., Lang, J. H., & Zhu, L. (2018). Diagnosis and Treatment of Rudimentary Horn Pregnancy: Analysis of Eleven Cases. *Chinese medical journal*, *131*(24), 3012–3014. https://doi.org/10.4103/0366-6999.247200 |
| 134 | Zhang, D. D., Gao, Y., Lang, J. H., & Zhu, L. (2018). Diagnosis and Treatment of Rudimentary Horn Pregnancy: Analysis of Eleven Cases. *Chinese medical journal*, *131*(24), 3012–3014. https://doi.org/10.4103/0366-6999.247200 |
| 135 | Zhang, D. D., Gao, Y., Lang, J. H., & Zhu, L. (2018). Diagnosis and Treatment of Rudimentary Horn Pregnancy: Analysis of Eleven Cases. *Chinese medical journal*, *131*(24), 3012–3014. https://doi.org/10.4103/0366-6999.247200 |
| 136 | Grover, V., Dhall, K., Dhall, G. I., & Dogra, M. (1986). Problems of rudimentary horn pregnancy. *Gynecologic and obstetric investigation*, *22*(1), 52–55. https://doi.org/10.1159/000298890 |
| 137 | Grover, V., Dhall, K., Dhall, G. I., & Dogra, M. (1986). Problems of rudimentary horn pregnancy. *Gynecologic and obstetric investigation*, *22*(1), 52–55. https://doi.org/10.1159/000298890 |
| 138 | Lim, J. S., & Tan, P. K. (2012). An unusual case of rudimentary uterine horn ectopic pregnancy. *Annals of the Academy of Medicine, Singapore*, *41*(2), 94–95. |
| 139 | Ponniah, R., Battina, S., & Battina, S. (2025). Pregnancy in a Non-Communicating Rudimentary Uterine Horn Managed With Laparoscopy in the First Trimester. *Journal of minimally invasive gynecology*, *32*(9), 753–754. https://doi.org/10.1016/j.jmig.2024.12.020 |
| 140 | Lata, I., Kapoor, D., Agarwal, S., & Niyaz, Z. (2013). Ruptured rudimentary horn pregnancy misdiagnosed as ruptured pseudo aneurysm internal iliac artery. *International journal of critical illness and injury science*, *3*(4), 284–285. https://doi.org/10.4103/2229-5151.124172 |
| 141 | Munck, D. F., Markauskas, A., Lamont, R. F., & Jørgensen, J. S. (2013). Pregnancy in a non-communicating rudimentary uterine horn in an obese woman. *Acta obstetricia et gynecologica Scandinavica*, *92*(7), 869. https://doi.org/10.1111/aogs.12098 |
| 142 | Hirose, I., Harada, K., Kuroda, R., Ishii, Y., Nakajima, M., Kamei, Y., Takazawa, Y., & Yoshida, K. (2013). An autopsy report on a ruptured rudimentary horn (uterine anomaly) with ectopic pregnancy. *Forensic science international*, *224*(1-3), e4–e6. https://doi.org/10.1016/j.forsciint.2012.10.019 |
| 143 | Yadav, S. K., Yadav, I., Jyoti, S., Lama, P., & Yadav, R. (2024). Primi at 16 weeks and 5 days of Gestation with Hypovolemic Shock Secondary to Ruptured Rudimentary Horn Ectopic Pregnancy: A Case Report. *JNMA; journal of the Nepal Medical Association*, *62*(274), 404–406. https://doi.org/10.31729/jnma.8617 |
| 144 | Alrawashdeh, M. M., & Alkazaleh, F. (2022). Successful Laparoscopic Management of Non-communicating Rudimentary Horn Pregnancy. *Cureus*, *14*(7), e27268. https://doi.org/10.7759/cureus.27268 |
| 145 | Okonta, P. I., Abedi, H., Ajuyah, C., & Omo-Aghoja, L. (2009). Pregnancy in a noncommunicating rudimentary horn of a unicornuate uterus: a case report. *Cases journal*, *2*, 6624. https://doi.org/10.1186/1757-1626-2-6624 |
| 146 | Kaur, J., Ramli, R., & Abd Kadir, N. J. (2025). Rudimentary horn pregnancy presenting with imminent uterine rupture. *BMJ case reports*, *18*(1), e264044. https://doi.org/10.1136/bcr-2024-264044 |
| 147 | Houmaid, H., & Hilali, A. (2021). Rupture of Rudimentary Horn Pregnancy at 16 Weeks of Gestation. *Case reports in obstetrics and gynecology*, *2021*, 8829053. https://doi.org/10.1155/2021/8829053 |
| 148 | Rajbhandary, S., Das, A., Rai, M., & Sah, A. K. (2020). Rupture of Non-communicating Rudimentary Horn Pregnancy at 15 Weeks with Previous Normal Pregnancies: A Case Report. *JNMA; journal of the Nepal Medical Association*, *58*(228), 614–617. https://doi.org/10.31729/jnma.5104 |
| 149 | Singh, N., Singh, U., & Verma, M. L. (2013). Ruptured bicornuate uterus mimicking ectopic pregnancy: A case report. *The journal of obstetrics and gynaecology research*, *39*(1), 364–366. https://doi.org/10.1111/j.1447-0756.2012.01914.x |
| 150 | Fouelifack, F. Y., Fouogue, J. T., Messi, J. O., Kamga, D. T., Fouedjio, J. H., & Sando, Z. (2014). Spontaneous second-trimester ruptured pregnancy of rudimentary horn: a case report in Yaounde, Cameroon. *The Pan African medical journal*, *18*, 86. https://doi.org/10.11604/pamj.2014.18.86.4579 |
| 151 | Amer, W. M., & Altraigey, A. (2020). A triplet's ectopic pregnancy in a non-communicating rudimentary horn and spontaneous rupture. *Ginekologia polska*, *91*(9), 569–570. https://doi.org/10.5603/GP.2020.0089 |
| 152 | Zeqiri, F., Paçarada, M., Kongjeli, N., Zeqiri, V., Kongjeli, G., & Krasniqi, B. (2010). Ruptured rudimentary horn pregnancy at sixteen weeks. *Journal of the Turkish German Gynecological Association*, *11*(3), 165–167. https://doi.org/10.5152/jtgga.2010.28 |
| 153 | Babu, K. M., & De, J. K. (2007). Rupture of Rudimentary Horn Pregnancy. *Medical journal, Armed Forces India*, *63*(1), 75–76. https://doi.org/10.1016/S0377-1237(07)80119-5 |
| 154 | Zhang, D. D., Gao, Y., Lang, J. H., & Zhu, L. (2018). Diagnosis and Treatment of Rudimentary Horn Pregnancy: Analysis of Eleven Cases. *Chinese medical journal*, *131*(24), 3012–3014. https://doi.org/10.4103/0366-6999.247200 |
| 155 | Zhang, D. D., Gao, Y., Lang, J. H., & Zhu, L. (2018). Diagnosis and Treatment of Rudimentary Horn Pregnancy: Analysis of Eleven Cases. *Chinese medical journal*, *131*(24), 3012–3014. https://doi.org/10.4103/0366-6999.247200 |
| 156 | Wang, L., Ren, S., Zhang, Y., Liu, W., & Shi, W. (2022). Non-communicated rudimentary uterine horn pregnancy and uterine rupture: A case report. *SAGE open medical case reports*, *10*, 2050313X221104034. https://doi.org/10.1177/2050313X221104034 |
| 157 | Matsubara, S., Saito, Y., Usui, R., & Takei, Y. (2011). Failure of mid-trimester pregnancy termination: ruptured rudimentary uterine horn pregnancy. *The journal of obstetrics and gynaecology research*, *37*(7), 967–968. https://doi.org/10.1111/j.1447-0756.2011.01656.x |
| 158 | Hafizi, L., & Ghomian, N. (2019). Twin pregnancy in the unicornuate uterus and non-communicating rudimentary horn: A case report. *International journal of reproductive biomedicine*, *17*(1), 67–70. https://doi.org/10.18502/ijrm.v17i1.3822 |
| 159 | Thakur, S., Sood, A., & Sharma, C. (2012). Ruptured noncommunicating rudimentary horn pregnancy at 19 weeks with previous cesarean delivery: a case report. *Case reports in obstetrics and gynecology*, *2012*, 308476. https://doi.org/10.1155/2012/308476 |
| 160 | Lauritsen, M. P., Larsen, E., & Johansen, M. (2010). Second trimester pregnancy in a rudimentary uterine horn. *Acta obstetricia et gynecologica Scandinavica*, *89*(8), 1111–1112. https://doi.org/10.3109/00016349.2010.497836 |
| 161 | Gülen, B., Serinken, M., Sonmez, E., Akpinar, G., & Söğüt, Ö. (2015). Rudimentary horn pregnancy mimicking an acute abdomen in the emergency department. *Journal of emergencies, trauma, and shock*, *8*(4), 237–238. https://doi.org/10.4103/0974-2700.166737 |
| 162 | Wang, Y., Yu, F., & Zeng, L. Q. (2015). Ectopic Pregnancy in Uncommon Implantation Sites: Intramural Pregnancy and Rudimentary Horn Pregnancy. *Case reports in obstetrics and gynecology*, *2015*, 536498. https://doi.org/10.1155/2015/536498 |
| 163 | Al Abbas, D. M., Aman, F. S., Almuhaimeed, R. S., & Almadeh, Z. M. (2024). Ruptured Ectopic Pregnancy in a Non-communicating Rudimentary Horn at 18 Weeks of Gestation. *Cureus*, *16*(12), e76199. https://doi.org/10.7759/cureus.76199 |
| 164 | Fitzmaurice, L. E., Ehsanipoor, R. M., & Porto, M. (2010). Rudimentary horn pregnancy with herniation into the main uterine cavity. *American journal of obstetrics and gynecology*, *202*(3), e5–e6. https://doi.org/10.1016/j.ajog.2009.12.011 |
| 165 | Hamet, B., Hoeffel, C., Fague, V., Lucot, J. P., Pagès-Bouic, E., Rousset, P., Graesslin, O., Bazot, M., & Poncelet, E. (2022). Pregnancy in a rudimentary horn: multicenter's MRI features of a rare condition. *Abdominal radiology (New York)*, *47*(12), 4195–4204. https://doi.org/10.1007/s00261-022-03658-3 |
| 166 | Hemlatha, A. L., Divya, P., Bharathi, M., & Parshwanath, H. A. (2005). Ectopic pregnancy unusually occuring in rudimentary uterine horn--report of two cases. *Indian journal of pathology & microbiology*, *48*(2), 245–247. |
| 167 | Kanagal, D. V., & Hanumanalu, L. C. (2012). Ruptured rudimentary horn pregnancy at 25 weeks with previous vaginal delivery: a case report. *Case reports in obstetrics and gynecology*, *2012*, 985076. https://doi.org/10.1155/2012/985076 |
| 168 | Sarikaya, S., & Aybay, M. N. (2022). Uterine rupture of a patient with rudimentary horn pregnancy at 26th gestational weeks. *International journal of surgery case reports*, *94*, 107003. https://doi.org/10.1016/j.ijscr.2022.107003 |
| 169 | Nishi, H., Funayama, H., Fukumine, N., Yagishita, M., Nohira, T., Suzuki, Y., Isaka, K., & Takayama, M. (2003). Rupture of pregnant noncommunicating rudimentary uterine horn with fetal salvage: a case report. *Archives of gynecology and obstetrics*, *268*(3), 224–226. https://doi.org/10.1007/s00404-002-0310-y |
| 170 | Heinonen, P. K., & Aro, P. (1988). Rupture of pregnant noncommunicating uterine horn with fetal salvage. *European journal of obstetrics, gynecology, and reproductive biology*, *27*(3), 261–265. https://doi.org/10.1016/0028-2243(88)90131-1 |
| 171 | Munkholm Larsen, P., Hahn-Pedersen, J., & Lange, A. P. (1983). Pregnancy in a non-communicating, rudimentary uterine horn with a successful outcome. *Acta obstetricia et gynecologica Scandinavica*, *62*(1), 93–94. https://doi.org/10.3109/00016348309155769 |
| 172 | Pillai, S. A., Mathew, M., Ishrat, N., Kakaria, A., Qureshi, A., & Vaidyanathan, G. (2015). Ruptured Rudimentary Horn Pregnancy Diagnosed by Preoperative Magnetic Resonance Imaging Resulting in Fetal Salvage. *Sultan Qaboos University medical journal*, *15*(3), e429–e432. https://doi.org/10.18295/squmj.2015.15.03.021 |
| 173 | Elsayegh, A., & Nwosu, E. C. (1998). Rupture of pregnancy in the communicating rudimentary uterine horn at 34 weeks. *Human reproduction (Oxford, England)*, *13*(12), 3566–3568. https://doi.org/10.1093/humrep/13.12.3566 |
| 174 | Pannu, D., Bharti, R., Anand, H. P., & Sharma, M. (2016). Term Live Secondary Abdominal Pregnancy: A Case Report. *The Malaysian journal of medical sciences : MJMS*, *23*(5), 96–99. https://doi.org/10.21315/mjms2016.23.5.13 |
| 175 | Arslan, T., Bilgiç, E., Sentürk, M. B., & Yücel, N. (2009). Rudimentary uterine horn pregnancy: a mystery diagnosis. *Fertility and sterility*, *92*(6), 2037.e1–2037.e20373. https://doi.org/10.1016/j.fertnstert.2009.08.027 |
| 176 | Kawthalkar, A. S., Gawande, M. S., Jain, S. H., Joshi, S. A., Ghike, S. D., & Bhalerao, A. V. (2011). Rare case of live birth in a ruptured rudimentary horn pregnancy. *The journal of obstetrics and gynaecology research*, *37*(8), 1169–1172. https://doi.org/10.1111/j.1447-0756.2010.01497.x |
| 177 | SCHOLTZ M. (1951). A full-time pregnancy in a rudimentary horn of the uterus. *The Journal of obstetrics and gynaecology of the British Empire*, *58*(2), 293–296. https://doi.org/10.1111/j.1471-0528.1951.tb04914.x |
| 178 | Feteh, V. F., Dimala, C. A., Njim, T., & Fuka, B. (2016). Post term pregnancy in a non-communicating rudimentary horn of a unicornuate uterus. *BMC research notes*, *9*, 209. https://doi.org/10.1186/s13104-016-2013-7 |
| 179 | Kadan, Y., & Romano, S. (2008). Rudimentary horn pregnancy diagnosed by ultrasound and treated by laparoscopy--a case report and review of the literature. *Journal of minimally invasive gynecology*, *15*(5), 527–530. https://doi.org/10.1016/j.jmig.2008.05.010 |
| 180 | Tsuda, H., Fujino, Y., Umesaki, N., Yamamoto, K., & Ogita, S. (1994). Preoperative diagnosis of a rudimentary uterine horn. *European journal of obstetrics, gynecology, and reproductive biology*, *56*(2), 143–145. https://doi.org/10.1016/0028-2243(94)90273-9 |
| 181 | Buntugu, K., Ntumy, M., Ameh, E., & Obed, S. (2008). Rudimentary horn pregnancy: pre-rupture diagnosis and management. *Ghana medical journal*, *42*(2), 92–94. |
| 182 | Cutner, A., Saridogan, E., Hart, R., Pandya, P., & Creighton, S. (2004). Laparoscopic management of pregnancies occurring in non-communicating accessory uterine horns. *European journal of obstetrics, gynecology, and reproductive biology*, *113*(1), 106–109. https://doi.org/10.1016/j.ejogrb.2003.09.020 |
| 183 | Sharma, D., Usha, M. G., Gaikwad, R., & Sudha, S. (2011). Laparoscopic Resection of Unruptured Rudimentary Horn Pregnancy. *Journal of gynecological endoscopy and surgery*, *2*(2), 101–104. https://doi.org/10.4103/0974-1216.114161 |
| 184 | Vo, C. V., Dinh, T. V., & Hankins, G. D. (2003). Value of ultrasound in the early diagnosis of prerupture uterine horn pregnancy. A case report. *The Journal of reproductive medicine*, *48*(6), 471–473. |
| 185 | Kirschner, R., Löfstrand, T., & Mark, J. (1979). Pregnancy in a non-communicating, rudimentary uterine horn. A reason for failed therapeutic second trimester abortion. *Acta obstetricia et gynecologica Scandinavica*, *58*(5), 499–501. https://doi.org/10.3109/00016347909154077 |
| 186 | Bruand, M., Thubert, T., Winer, N., Gueudry, P., & Dochez, V. (2020). Rupture of Non-communicating Rudimentary Horn of Uterus at 12 Weeks' Gestation. *Cureus*, *12*(3), e7191. https://doi.org/10.7759/cureus.7191 |
| 187 | Srinivas, T., Kirschen, G. W., & Yazdy, G. M. (2024). Laparoscopic Management of a 12-Week Pregnancy Loss in a Rudimentary Uterine Horn. *Cureus*, *16*(6), e61677. https://doi.org/10.7759/cureus.61677 |
| 188 | Yoo, E. H., Chun, S. H., & Woo, B. H. (1999). Laparoscopic resection of a rudimentary horn pregnancy. *Acta obstetricia et gynecologica Scandinavica*, *78*(2), 167–168. |
| 189 | Lennox, G., Pantazi, S., Keunen, J., Van Mieghem, T., & Allen, L. (2013). Minimally invasive surgical management of a second trimester pregnancy in a rudimentary uterine horn. *Journal of obstetrics and gynaecology Canada : JOGC = Journal d'obstetrique et gynecologie du Canada : JOGC*, *35*(5), 468–472. https://doi.org/10.1016/S1701-2163(15)30938-5 |
| 190 | Daaloul, W., Ouerdiane, N., Khoudaier, M., Masmoudi, A., Ennine, I., Ben Hamouda, S., Bouguerra, B., & Sfar, R. (2012). Stopped pregnancy in a rudimentary horn at 12 week gestation. *La Tunisie medicale*, *90*(6), 485–487. |
| 191 | Gagnon, A. L., Galerneau, F., & Williams, K. (1998). Twin pregnancy with one fetus in a rudimentary horn: a case report of a surviving twin. *British journal of obstetrics and gynaecology*, *105*(12), 1326–1328. https://doi.org/10.1111/j.1471-0528.1998.tb10015.x |
| 192 | Brady, P. C., Molina, R. L., Muto, M. G., Stapp, B., & Srouji, S. S. (2018). Diagnosis and management of a heterotopic pregnancy and ruptured rudimentary uterine horn. *Fertility research and practice*, *4*, 6. https://doi.org/10.1186/s40738-018-0051-7 |
| 193 | Hamet, B., Hoeffel, C., Fague, V., Lucot, J. P., Pagès-Bouic, E., Rousset, P., Graesslin, O., Bazot, M., & Poncelet, E. (2022). Pregnancy in a rudimentary horn: multicenter's MRI features of a rare condition. *Abdominal radiology (New York)*, *47*(12), 4195–4204. https://doi.org/10.1007/s00261-022-03658-3 |
| 194 | Hamet, B., Hoeffel, C., Fague, V., Lucot, J. P., Pagès-Bouic, E., Rousset, P., Graesslin, O., Bazot, M., & Poncelet, E. (2022). Pregnancy in a rudimentary horn: multicenter's MRI features of a rare condition. *Abdominal radiology (New York)*, *47*(12), 4195–4204. https://doi.org/10.1007/s00261-022-03658-3 |
| 195 | Siwatch, S., Mehra, R., Pandher, D. K., & Huria, A. (2013). Rudimentary horn pregnancy: a 10-year experience and review of literature. *Archives of gynecology and obstetrics*, *287*(4), 687–695. https://doi.org/10.1007/s00404-012-2625-7 |
| 196 | Siwatch, S., Mehra, R., Pandher, D. K., & Huria, A. (2013). Rudimentary horn pregnancy: a 10-year experience and review of literature. *Archives of gynecology and obstetrics*, *287*(4), 687–695. https://doi.org/10.1007/s00404-012-2625-7 |
| 197 | Chatziioannidou, K., Fehlmann, A., & Dubuisson, J. (2020). Case Report: Laparoscopic Management of an Ectopic Pregnancy in a Rudimentary Non-communicating Uterine Horn. *Frontiers in surgery*, *7*, 582954. https://doi.org/10.3389/fsurg.2020.582954 |
| 198 | Kapoor, S., Gardner, F. J., de Chazal, R., & Brown, L. J. (2008). Ruptured rudimentary horn and TRAP syndrome. *Journal of obstetrics and gynaecology : the journal of the Institute of Obstetrics and Gynaecology*, *28*(3), 358–359. https://doi.org/10.1080/01443610802058874 |
| 199 | Hamet, B., Hoeffel, C., Fague, V., Lucot, J. P., Pagès-Bouic, E., Rousset, P., Graesslin, O., Bazot, M., & Poncelet, E. (2022). Pregnancy in a rudimentary horn: multicenter's MRI features of a rare condition. *Abdominal radiology (New York)*, *47*(12), 4195–4204. https://doi.org/10.1007/s00261-022-03658-3 |
| 200 | Abd El-Halim, D., & Torky, H. A. (2017). Pregnancy in a non-communicating rudimentary horn: a cause of failed medical and surgical management of second trimester pregnancy loss. *The European journal of contraception & reproductive health care : the official journal of the European Society of Contraception*, *22*(5), 391–392. https://doi.org/10.1080/13625187.2017.1312326 |
| 201 | Ozeren, S., Caliskan, E., Corakci, A., Ozkan, S., & Demirci, A. (2004). Magnetic resonance imaging and angiography for the prerupture diagnosis of rudimentary uterine horn pregnancy. *Acta radiologica (Stockholm, Sweden : 1987)*, *45*(8), 878–881. https://doi.org/10.1080/02841850410008252 |
| 202 | Hamet, B., Hoeffel, C., Fague, V., Lucot, J. P., Pagès-Bouic, E., Rousset, P., Graesslin, O., Bazot, M., & Poncelet, E. (2022). Pregnancy in a rudimentary horn: multicenter's MRI features of a rare condition. *Abdominal radiology (New York)*, *47*(12), 4195–4204. https://doi.org/10.1007/s00261-022-03658-3 |
| 203 | Pineda Rivas, M., Rattray, D., Suchet, I., & Thiel, J. (2015). Laparoscopic Resection of 16 Week Pregnancy in a Rudimentary Uterine Horn. *Journal of minimally invasive gynecology*, *22*(6S), S126. https://doi.org/10.1016/j.jmig.2015.08.383 |
| 204 | Cash, R. L., Rahmani, R., & Herer, E. R. (2006). First trimester screening aids in the diagnosis and management of an ectopic pregnancy in a noncommunicating uterine horn. *Journal of clinical ultrasound : JCU*, *34*(9), 446–449. https://doi.org/10.1002/jcu.20260 |
| 205 | Zhang, S., Lamari, A., Ferris, E., & Maseelall, P. (2022). Fertility after treatment of a noncommunicating rudimentary horn pregnancy: A case report. *Case reports in women's health*, *35*, e00429. https://doi.org/10.1016/j.crwh.2022.e00429 |
| 206 | Hamet, B., Hoeffel, C., Fague, V., Lucot, J. P., Pagès-Bouic, E., Rousset, P., Graesslin, O., Bazot, M., & Poncelet, E. (2022). Pregnancy in a rudimentary horn: multicenter's MRI features of a rare condition. *Abdominal radiology (New York)*, *47*(12), 4195–4204. https://doi.org/10.1007/s00261-022-03658-3 |
| 207 | Shahid, A., Olowu, O., Kandasamy, G., O'Donnell, C., & Odejinmi, F. (2010). Laparoscopic management of a 16-week ruptured rudimentary horn pregnancy: a case and literature review. *Archives of gynecology and obstetrics*, *282*(2), 121–125. https://doi.org/10.1007/s00404-009-1212-z |
| 208 | Hemlatha, A. L., Divya, P., Bharathi, M., & Parshwanath, H. A. (2005). Ectopic pregnancy unusually occuring in rudimentary uterine horn--report of two cases. *Indian journal of pathology & microbiology*, *48*(2), 245–247. |
| 209 | Yildirim, D., Turkgeldi, L. S., Tekiner, N., Seckin, K. D., & Yucel, B. (2017). A case of rudimentary horn pregnancy diagnosed after failed attempts at pregnancy termination. *Nigerian journal of clinical practice*, *20*(1), 111–114. https://doi.org/10.4103/1119-3077.187317 |
| 210 | Siwatch, S., Mehra, R., Pandher, D. K., & Huria, A. (2013). Rudimentary horn pregnancy: a 10-year experience and review of literature. *Archives of gynecology and obstetrics*, *287*(4), 687–695. https://doi.org/10.1007/s00404-012-2625-7 |
| 211 | Abreu, R., Barros, S., Jardim, O., & Morais, C. (2010). Failure of prostaglandin induction labor in a Müllerian abnormality. *Archives of gynecology and obstetrics*, *282*(2), 143–147. https://doi.org/10.1007/s00404-010-1361-0 |
| 212 | LATTO, D., & NORMAN, R. (1950). Pregnancy in a rudimentary horn of a bicornuate uterus. *British medical journal*, *2*(4685), 926–927. https://doi.org/10.1136/bmj.2.4685.926 |
| 213 | Nathan, H., & Sornum, A. (2013). Diagnosis and management of a ruptured rudimentary horn pregnancy in a low-resource setting. *BMJ case reports*, *2013*, bcr2013009957. https://doi.org/10.1136/bcr-2013-009957 |
| 214 | Kuşcu, N. K., Laçin, S., Kartal, O., & Koyuncu, F. (2002). Rupture of rudimentary horn pregnancy at the 15th week of gestation: a case report. *European journal of obstetrics, gynecology, and reproductive biology*, *102*(2), 209–210. https://doi.org/10.1016/s0301-2115(01)00600-5 |
| 215 | REYNER, F. C., & WETCHLER, B. B. (1955). Rupture of rudimentary horn of uterus bicornis unicollis with pregnancy of 41/2 months. *American journal of obstetrics and gynecology*, *69*(1), 197–199. https://doi.org/10.1016/s0002-9378(16)37931-5 |
| 216 | Dhar H. (2008). Rupture of non-communicating rudimentary uterine horn pregnancy. *Journal of the College of Physicians and Surgeons--Pakistan : JCPSP*, *18*(1), 53–54. |
| 217 | Bhattachary, T. K., & Sengupta, P. (2005). Rudimentary Horn Pregnancy. *Medical journal, Armed Forces India*, *61*(4), 377–378. https://doi.org/10.1016/S0377-1237(05)80073-5 |
| 218 | Jayasinghe, Y., Rane, A., Stalewski, H., & Grover, S. (2005). The presentation and early diagnosis of the rudimentary uterine horn. *Obstetrics and gynecology*, *105*(6), 1456–1467. https://doi.org/10.1097/01.AOG.0000161321.94364.56 |
| 219 | Rathod, S., & Samal, S. K. (2015). A rare case of heterotopic pregnancy with ruptured left rudimentary horn pregnancy. *Journal of clinical and diagnostic research : JCDR*, *9*(3), QD03–QD4. https://doi.org/10.7860/JCDR/2015/10677.5639 |
| 220 | Accordino, F., Pintucci, A., Manni, M. U., Meregalli, V., & Locatelli, A. (2019). Re to: Tale of rudimentary horn pregnancy and literature review. *The journal of maternal-fetal & neonatal medicine : the official journal of the European Association of Perinatal Medicine, the Federation of Asia and Oceania Perinatal Societies, the International Society of Perinatal Obstetricians*, *32*(16), 2778–2779. https://doi.org/10.1080/14767058.2018.1448775 |
| 221 | Kukreti, M., Singhal, V. P., Kukreti, R., & Prakash, A. (2004). Pregnancy in a rupturing non-communicating rudimentary horn masquerading as epigastric pain. *The Australian & New Zealand journal of obstetrics & gynaecology*, *44*(5), 470–472. https://doi.org/10.1111/j.1479-828X.2004.00276.x |
| 222 | Abbasi, Z., Das, S., Thapa, U., Aryal, S., & Mughal, S. (2019). Ruptured Ectopic Pregnancy in an Accessory Horn of Uterus: A Case Report. *Cureus*, *11*(12), e6436. https://doi.org/10.7759/cureus.6436 |
| 223 | Hussain, A., Jawaid, H., Faisal, N., Shah, N., & Kamal, N. S. (2018). Ruptured Rudimentary Horn Pregnancy Revealed on Emergency Laparotomy: A Case of Primigravida Presenting in a Developing Country. *Cureus*, *10*(5), e2591. https://doi.org/10.7759/cureus.2591 |
| 224 | Hassan, C. H., Karim, A. K., Ismail, N. A., & Omar, M. H. (2011). Case report of ruptured non-communicating right rudimentary horn pregnancy: an acute emergency. *Acta medica (Hradec Kralove)*, *54*(3), 125–126. https://doi.org/10.14712/18059694.2016.34 |
| 225 | Has, R., Ermis, H., & Yildirim, A. (2000). A malformed fetus in a rudimentary uterine horn pregnancy. *Ultrasound in obstetrics & gynecology : the official journal of the International Society of Ultrasound in Obstetrics and Gynecology*, *16*(2), 200–202. https://doi.org/10.1046/j.1469-0705.2000.00207.x |
| 226 | Bilgin, T., Demir, V., & Esmer, A. (1992). Rupture of noncommunicating rudimentary uterine horn pregnancy. *International journal of gynaecology and obstetrics: the official organ of the International Federation of Gynaecology and Obstetrics*, *38*(2), 126–128. https://doi.org/10.1016/0020-7292(92)90049-o |
| 227 | Dhar H. (2012). Ruptured rudimentary horn at 22 weeks. *Nigerian medical journal : journal of the Nigeria Medical Association*, *53*(3), 175–177. https://doi.org/10.4103/0300-1652.104391 |
| 228 | Nwosu, U. C., & Thatcher, S. (1993). Pregnancy in a non-communicating uterine horn mimicking incarceration with sacculation of a retroflexed uterus. *Acta obstetricia et gynecologica Scandinavica*, *72*(7), 580–582. https://doi.org/10.3109/00016349309058168 |
| 229 | Tufail, A., & Hashmi, H. A. (2007). Ruptured ectopic pregnancy in rudimentary horn of the uterus. *Journal of the College of Physicians and Surgeons--Pakistan : JCPSP*, *17*(2), 105–106. |
| 230 | Oral, B., Güney, M., Ozsoy, M., & Sönal, S. (2001). Placenta accreta associated with a ruptured pregnant rudimentary uterine horn. Case report and review of the literature. *Archives of gynecology and obstetrics*, *265*(2), 100–102. https://doi.org/10.1007/s004040000140 |
| 231 | Başbuğ, M., Soyuer, I., & Aygen, E. (1997). Placenta accreta associated with rupture of a rudimentary horn pregnancy. *International journal of gynaecology and obstetrics: the official organ of the International Federation of Gynaecology and Obstetrics*, *57*(2), 199–201. https://doi.org/10.1016/s0020-7292(97)02895-6 |
| 232 | Ghotra, M. K., Jr, Joshi, B., & Bhutani, S. (2021). Ruptured Rudimentary Horn Pregnancy: Delayed Diagnosis. *Cureus*, *13*(6), e15873. https://doi.org/10.7759/cureus.15873 |
| 233 | Kaveh, M., Mehdizadeh Kashi, A., Sadegi, K., & Forghani, F. (2018). Pregnancy in Non-Communicating Rudimentary Horn of A Unicornuate Uterus. *International journal of fertility & sterility*, *11*(4), 318–320. https://doi.org/10.22074/ijfs.2018.5022 |
| 234 | Hamet, B., Hoeffel, C., Fague, V., Lucot, J. P., Pagès-Bouic, E., Rousset, P., Graesslin, O., Bazot, M., & Poncelet, E. (2022). Pregnancy in a rudimentary horn: multicenter's MRI features of a rare condition. *Abdominal radiology (New York)*, *47*(12), 4195–4204. https://doi.org/10.1007/s00261-022-03658-3 |
| 235 | Contreras, K. R., Rothenberg, J. M., Kominiarek, M. A., & Raff, G. J. (2008). Hand-assisted laparoscopic management of a midtrimester rudimentary horn pregnancy with placenta increta: a case report and literature review. *Journal of minimally invasive gynecology*, *15*(5), 644–648. https://doi.org/10.1016/j.jmig.2008.06.008 |
| 236 | Jain, R., Gami, N., Puri, M., & Trivedi, S. (2010). A rare case of intact rudimentary horn pregnancy presenting as hemoperitoneum. *Journal of human reproductive sciences*, *3*(2), 113–115. https://doi.org/10.4103/0974-1208.69335 |
| 237 | Jarrell, J., Effer, S. B., & Mohide, P. T. (1977). Pregnancy in a rudimentary horn with fetal salvage. *American journal of obstetrics and gynecology*, *127*(6), 676–677. https://doi.org/10.1016/0002-9378(77)90376-3 |
| 238 | McCarthy E. (1999). A case report and review of pregnancies in rudimentary noncommunicating uterine horns. *The Australian & New Zealand journal of obstetrics & gynaecology*, *39*(2), 188–190. https://doi.org/10.1111/j.1479-828x.1999.tb03369.x |
| 239 | Gonçalves, E., Prata, J. P., Ferreira, S., Abreu, R., Mesquita, J., Carvalho, A., & Pinheiro, P. (2013). An unexpected near term pregnancy in a rudimentary uterine horn. *Case reports in obstetrics and gynecology*, *2013*, 307828. https://doi.org/10.1155/2013/307828 |
| 240 | Singh, P., Gupta, R., Das, B., Bajaj, S. K., & Misra, R. (2015). Midtrimester spontaneous torsion of unruptured gravid rudimentary horn: Presurgical diagnosis on magnetic resonance imaging. *The journal of obstetrics and gynaecology research*, *41*(9), 1478–1482. https://doi.org/10.1111/jog.12722 |
| 241 | Ambusaidi, Q., & Jha, C. (2014). Pregnancy in the Rudimentary Uterine Horn: Case report of an unusual presentation. *Sultan Qaboos University medical journal*, *14*(1), e134–e138. https://doi.org/10.12816/0003349 |
| 242 | Siwatch, S., Mehra, R., Pandher, D. K., & Huria, A. (2013). Rudimentary horn pregnancy: a 10-year experience and review of literature. *Archives of gynecology and obstetrics*, *287*(4), 687–695. https://doi.org/10.1007/s00404-012-2625-7 |
| 243 | Abreu, R., Barros, S., Jardim, O., & Morais, C. (2010). Failure of prostaglandin induction labor in a Müllerian abnormality. *Archives of gynecology and obstetrics*, *282*(2), 143–147. https://doi.org/10.1007/s00404-010-1361-0 |
| 244 | Ross, M. E., Scott, S., Behbakht, K., & Harper, T. (2020). Spontaneous dichorionic-diamniotic twins in a noncommunicating uterine horn: A case report. *Case reports in women's health*, *26*, e00177. https://doi.org/10.1016/j.crwh.2020.e00177 |
| 245 | Sutkin, G., & Jazayeri, A. (2003). Diagnosis of a rudimentary uterine horn in pregnancy. *Journal of ultrasound in medicine : official journal of the American Institute of Ultrasound in Medicine*, *22*(9), 985–988. https://doi.org/10.7863/jum.2003.22.9.985 |
| 246 | Letterie, G. S., Murray, J. E., & Samlaska, C. P. (1993). Failure of prostaglandin induction in an obstructive müllerian abnormality. *American journal of obstetrics and gynecology*, *169*(1), 207–209. https://doi.org/10.1016/0002-9378(93)90167-h |
| 247 | Seoud, M. A., Khalil, A. M., Abdel-Karim, F. W., & Suidan, J. S. (1989). Pregnancy in a non-communication rudimentary uterine horn. *International journal of gynaecology and obstetrics: the official organ of the International Federation of Gynaecology and Obstetrics*, *28*(3), 275–278. https://doi.org/10.1016/0020-7292(89)90730-3 |
| 248 | Coakley, K. E., Yang, T. B., & Chung, J. H. (2021). Periviable delivery of a pregnancy in a rudimentary uterine horn: A case report. *Case reports in women's health*, *32*, e00346. https://doi.org/10.1016/j.crwh.2021.e00346 |
| 249 | Daskalakis, G., Pilalis, A., Lykeridou, K., & Antsaklis, A. (2002). Rupture of noncommunicating rudimentary uterine horn pregnancy. *Obstetrics and gynecology*, *100*(5 Pt 2), 1108–1110. https://doi.org/10.1016/s0029-7844(02)02153-1 |
| 250 | Tsai, S., Uzelac, A., Lindheim, S. R., & Pereira, N. (2024). Surgical management of an ectopic pregnancy in the setting of an unexpected Müllerian anomaly: intraoperative and postoperative implications. *Fertility and sterility*, *122*(5), 951–953. https://doi.org/10.1016/j.fertnstert.2024.07.036 |
