# Supplementary material for: Robotic resection of a rudimentary horn pregnancy with the Da Vinci system: first case and systematic review of published cases in the 21st century
Source: Front Surg. 2026 May 18;13:1757761. doi: 10.3389/fsurg.2026.1757761 (PMC13223003; doi:10.3389/fsurg.2026.1757761)
Supplement: Supplementary file 3 [file Table3.docx]

**Identification of studies via other methods**

**Identification of studies via databases and registers**

Records identified from:

Websites (n = 0)

Organisations (n = 0)

Citation searching (n = 0)

etc.

Records removed *before screening*:

Duplicate records removed (n = 0)

Records marked as ineligible by automation tools (n = 0)

Records removed for other reasons (n = 0)

Records identified from:

Databases (n = 242)

Registers (n = 0)

**Identification**

Records screened

(n = 242)

Records excluded

(n = 18)

Reports not retrieved

(n = 0)

Reports sought for retrieval

(n = 0)

Reports sought for retrieval

(n = 224)

Reports not retrieved

(n = 216)

**Screening**

Reports assessed for eligibility

(n = 0)

Reports excluded:

(n = 0)

Reports assessed for eligibility

(n = 216)

Reports excluded:

- Rudimentary Horn without pregnancy: 34

- Communicating rudimentary Horn or other anomalies: 12

- Other ectopic pregnancy: 7

- Others: 8

Studies included in review

(n = 163)

Reports of included studies

(n = 0)

**Included**

Source: Page MJ, et al. BMJ 2021;372:n71. doi: 10.1136/bmj.n71.

This work is licensed under CC BY 4.0. To view a copy of this license, visit <https://creativecommons.org/licenses/by/4.0/>
